# Macrophage pyroptosis inhibition alleviates postinjury neointimal formation and vascular restenosis

**DOI:** 10.1186/s12967-026-07777-z

**Published:** 2026-02-03

**Authors:** Zaixiong Ji, Meijuan He, Hong Wu, Shixiong Chen, Suhe Wang, Xiaorui Yin, Han Wang

**Affiliations:** 1 Department of Interventional Radiology, Shanghai General Hospital, Shanghai Jiao Tong University School of Medicine, Shanghai, 200080 P. R. China; 2 National Center for Translational Medicine (Shanghai), Shanghai, 201620 P. R. China; 3 R & D Center of Medical Artificial Intelligence and Medical Engineering, Haining Rd.100, Shanghai General Hospital, Shanghai, 200080 P. R. China; 4 Department of Internal Medicine, Michigan Nanotechnology Institute for Medicine and Biological Sciences, University of Michigan, 109 Zina Pitcher Place, Ann Arbor, MI 48109 USA; 5 Jiading Branch, Shanghai General Hospital, Shanghai, 201812 P. R. China

**Keywords:** Vascular injury, Restenosis, Pyroptosis, Disulfiram, Balloon coating

## Abstract

**Background:**

Clinically, restenosis remains the leading cause of long-term failure following endovascular interventions for cardiovascular diseases. However, the underlying pathogenesis of restenosis is not yet fully elucidated. Current therapeutic devices, such as drug-coated balloons or stents, are typically loaded with non-specific antiproliferative agents, whose broad cytotoxic effects may exacerbate vascular inflammation. This study investigates the mechanism of restenosis following endovascular interventions and develops a novel targeted therapeutic strategy based on the identified mechanism.

**Methods:**

Single-cell transcriptomic sequencing was employed to investigate cellular and gene changes in restenotic vessels. Macrophage-specific knockdown of GSDMD was performed, and disulfiram-coated balloon (DCB) is developed to deliver disulfiram (DSF) to the target vessel, blocking GSDMD-mediated pyroptosis. The effects of this strategy were validated through systematic in vivo and in vitro experiments.

**Results:**

Here, elevated expression levels of pyroptosis-related genes were observed in blood samples from patients with restenosis. Single-cell transcriptomic analysis revealed that macrophages were the key cell population undergoing pyroptosis in vessels with restenosis. Immunofluorescence co-staining of rat vessels demonstrated that GSDMD was localized in the macrophages within restenotic vessels. Immunoblot analysis further confirmed that GSDMD was in its activated state in restenotic vessels. Knockdown of specific macrophage GSDMD ameliorated inflammatory response, promoted vascular endothelial repair, and inhibited neointimal formation. To translate these mechanistic insights into therapy, the DCB was developed to enable localized delivery of DSF to the injured vessel wall, thereby effectively inhibiting GSDMD pore formation. Results from the rat carotid artery balloon injury model indicated that by targeting GSDMD, DCB inhibited macrophage pyroptosis and associated inflammation, promoting functional re-endothelialization, and reducing neointimal hyperplasia. Mechanistically, in vitro co-culture experiments demonstrated that GSDMD-mediated macrophage pyroptosis plays a key role in the differential regulation of vascular endothelial cells and smooth muscle cells by DSF.

**Conclusion:**

Collectively, our findings identify GSDMD as a novel and therapeutically relevant target in vascular restenosis. Based on this mechanism, DCB, which inhibits pyroptosis by targeting GSDMD, represents a promising therapeutic strategy for prevention of restenosis.

**Supplementary Information:**

The online version contains supplementary material available at 10.1186/s12967-026-07777-z.

## Highlights

Newly infiltrated macrophages undergo GSDMD-mediated pyroptosis in injured vessels after endovascular interventions.

GSDMD-mediated macrophage pyroptosis contributes to endothelial damage and neointimal formation.

The disulfiram-coated balloon (DCB) is specifically engineered for targeted delivery of disulfiram to the injured vessel wall, inhibiting GSDMD activation and pyroptotic pore formation.

DCB promotes functional re-endothelialization and suppresses neointimal hyperplasia in the injured vessel.

**Supplementary Information:**

The online version contains supplementary material available at 10.1186/s12967-026-07777-z.

## Introduction

Cardiovascular disease (CVD) is the leading cause of death worldwide and has become the primary threat to human health [1]. Endovascular intervention, which employs balloons or stents to dilate occluded vascular segments and restore blood flow, has become the preferred treatment for certain occlusive cardiovascular diseases caused by atherosclerosis, diabetes, hypertension, and other related conditions [2, 3]. However, vascular restenosis initiated by the disruption and dysfunction of the endothelial barrier, has emerged as a major drawback of endovascular intervention, severely affecting prognosis in patients and resulting in long-term treatment failure [4, 5]. The evolution of commercial drug coated balloons or stents has witnessed the development of device coatings with non-specific antiproliferative agents such as paclitaxel (PTX), which have shown some effectiveness for restenosis prevention in the short-term [6–11]. Nevertheless, the indiscriminate cytotoxicity of drug coatings with antiproliferative agents has led to exacerbated inflammation, delayed endothelial healing, and late-stage thrombosis (occurring beyond 36 months, after the limited elution period) [6, 12, 13]. Thus, the development of novel therapeutic strategies for vascular restenosis prevention is urgently needed.

Endovascular intervention inevitably causes collateral damage to the endothelial barrier and vessel wall, with intimal tearing leading to collagen exposure and sustained inflammation [14, 15]. The accumulation of macrophages in the damaged vascular wall is associated with chronic inflammation in restenotic vessels [16–22]. Inflammatory responses lead to early endothelial cell (EC) dysfunction following vascular injury, subsequently causing excessive proliferation of smooth muscle cells (SMC) [23]. Pyroptosis is a type of gasdermin protein-mediated inflammatory cell death that contributes to an exacerbation of inflammation [24]. Pyroptosis is associated with restenosis, as levels of interleukin (IL)-1β and IL-18 were significantly elevated in patients with restenosis compared to the control group [25–27]. Canonical inflammasomes, such as the nucleotide-binding oligomerization domain-like receptor protein 3 (NLRP3) inflammasome, activate caspase-1 [28]. Activated caspase-1 cleaves gasdermin D (GSDMD) into the N-terminal domain (GSDMD-NT), which forms membrane pores, enabling the release of pro-inflammatory cytokines IL-1β and IL-18 and inducing pyroptotic cell death, further exacerbating inflammation [29]. However, the role of pyroptosis in vascular restenosis is not fully understood.

GSDMD has been identified as the final common effector downstream of inflammasomes activation in inflammatory diseases and cancer, which opens new therapeutic avenues targeting pyroptosis [30, 31]. Inhibiting GSDMD will potentially curb inflammation induced by all inflammasomes activation triggered by any stimulus. Therefore, targeting GSDMD-mediated pyroptosis might provide a more comprehensive regulation of the inflammatory response compared to inhibiting just one specific inflammasome such as NLRP3 inflammasome, making it an especially attractive therapeutic for inflammatory diseases [32, 33]. Driven by a rising number of studies indicating the role of gasdermin protein in the pathogenesis of cardiovascular diseases and injuries, the antagonism of gasdermin protein is gaining increasing attention in vascular medicine [34–40]. However, the role of GSDMD in vascular restenosis remains unclear. If GSDMD-mediated pyroptosis promotes vascular restenosis, then new strategies devised to inhibit GSDMD based on this mechanism may exert more potent anti-restenosis effects.

Disulfiram (DSF) is an FDA-approved drug primarily used for the treatment of alcohol addiction [41]. Recent studies have shown that DSF is a pyroptosis inhibitor that directly blocks GSDMD pore formation [32]. Compared to inhibitors that target individual inflammasomes and neutralizing antibodies against pro-inflammatory cytokines, DSF targeting GSDMD pore formation may inhibit inflammation resulting from the activation of all inflammasomes, thereby exerting a more potent anti-inflammatory effect [32]. DSF has already demonstrated therapeutic effects in cardiovascular diseases [42–45]. However, there is currently no evidence regarding the potential of DSF and GSDMD-mediated pyroptosis inhibition in preventing restenosis in the setting of vascular drug-coated devices.

**Scheme 1:**
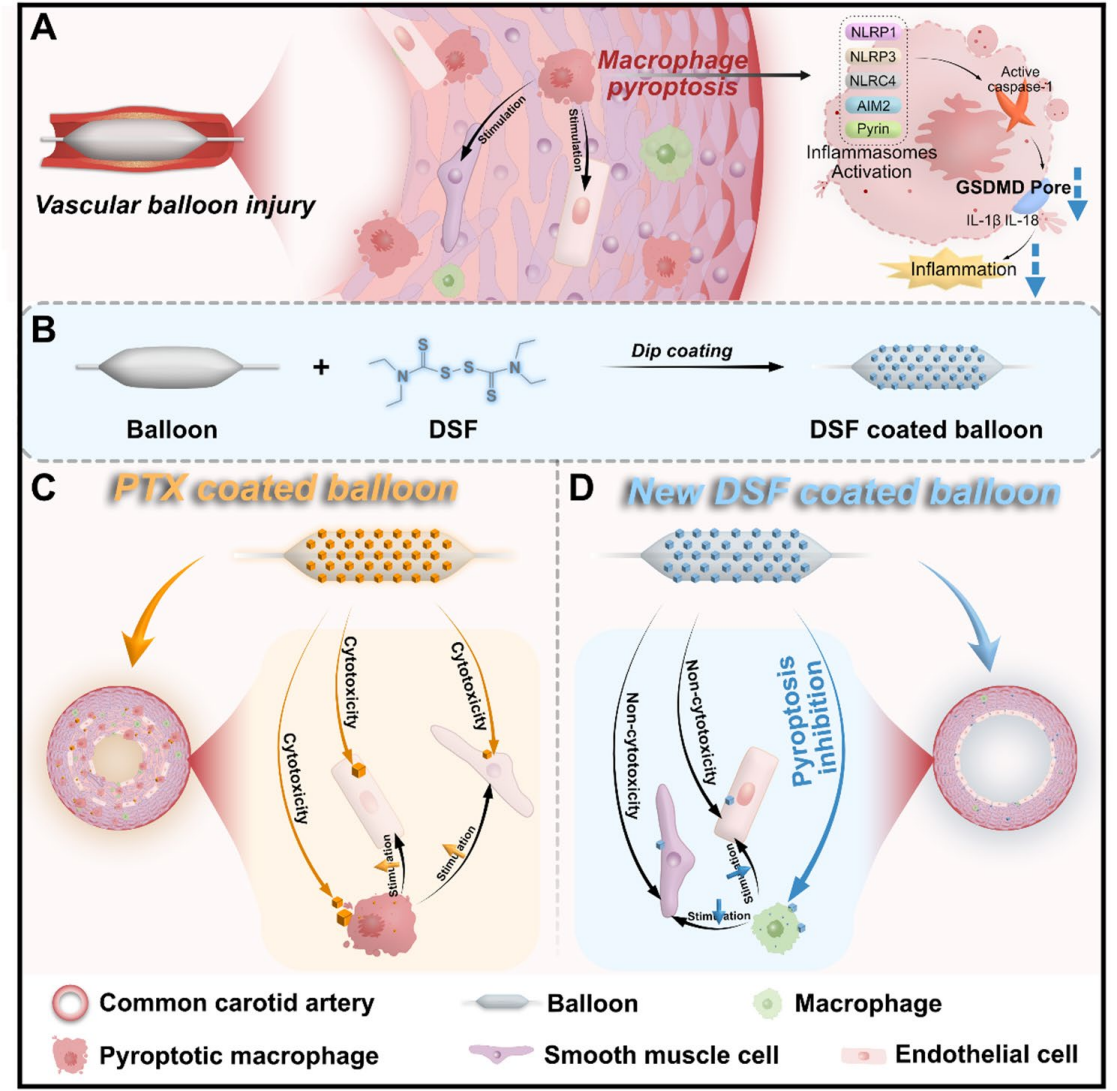
Schematic illustration of GSDMD as an anti-restenosis target and devised mechanism-based disulfiram-coated balloon (DCB) against restenosis. GSDMD-mediated macrophage pyroptosis contributes to vascular inflammation and restenosis after endovascular intervention (**A**). The customed DCB (**B**), with a favorable biosafety profile, by directly targeting GSDMD pore formation to inhibit pyroptosis, promoting functional endothelial repair and inhibiting neointimal hyperplasia (**D**), thereby superiorly preventing long-term vascular restenosis compared to paclitaxel (PTX)-coated balloon (**C**)

In this study, we discover that GSDMD-mediated macrophage pyroptosis induced by the activation of multiple inflammasomes promotes vascular inflammation and restenosis after endovascular interventions. Based on the mechanism, we design and test a DSF-coated balloon (DCB) to precisely deliver DSF to the injured vascular wall for restenosis prevention. The DCB consists of two parts with different functions: (I) The balloon, serving as a carrier to achieve precise delivery of DSF crystalline to the injured vascular wall, overcoming the issue of insufficient local dosage in traditional systemic administration, and avoiding systemic side effects caused by increased total dosage and the need for additional invasive treatments; (II) The DSF coating, loaded on the balloon surface in crystalline form, which can be easily transferred to the vascular wall through balloon dilation, locally inhibiting pyroptosis via sustained release and exerting protective effects against restenosis. The developed DCB, by directly targeting GSDMD pore formation to inhibit pyroptosis, accelerates the repair of functional endothelial cells and inhibits the excessive migration and proliferation of smooth muscle cells, providing a promising therapeutic alternative for restenosis prevention (Scheme 1).

## Results

### GSDMD-mediated macrophage pyroptosis in injured vessels after endovascular intervention contributes to endothelial damage and neointimal hyperplasia

**Fig. 1:**
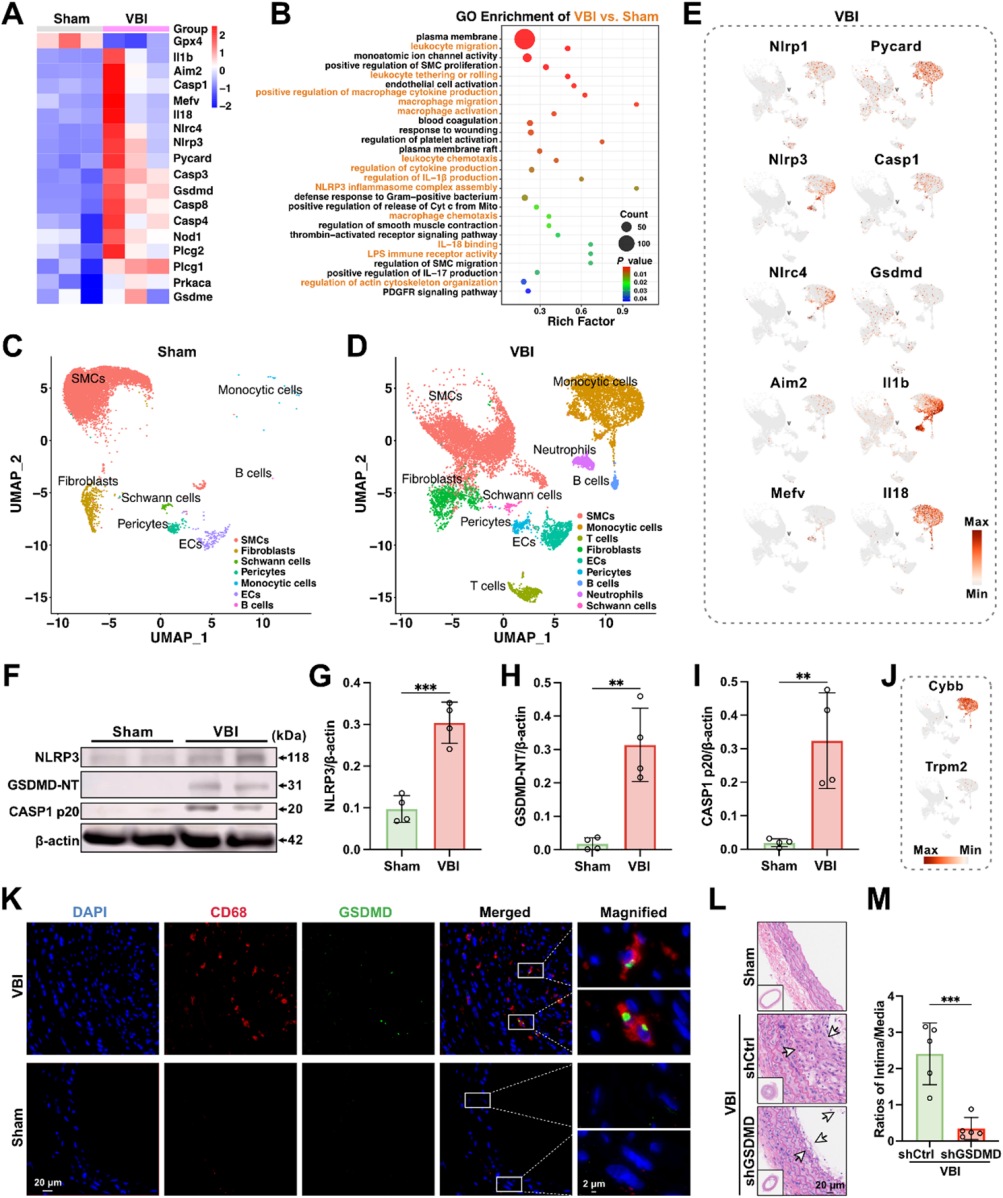
GSDMD-mediated macrophage pyroptosis in injured vessels after endovascular intervention contributes to endothelial damage and neointimal hyperplasia. (**A**) Heatmap showing genes related to pyroptotic inflammation in the Sham group and vascular balloon injury (VBI) group (*n* = 3). (**B**) Gene Ontology (GO) analysis of differentially expressed genes between the VBI group and the Sham group (*n* = 3). (**C** and **D**) Cells from the common carotid arteries of rats in the Sham group (C) and VBI group (**D**) were analyzed using scRNA-Seq. Visualization of clustering of arterial cells using a Uniform Manifold Approximation and Projection (UMAP) plot. (**E**) Biaxial scatter plots showing the distribution and expression of key genes involved in pyroptosis across all cell populations in the VBI group. (**F**-**I**) Western blotting analysis of left common carotid arteries from control (Sham) rats and VBI rats (**F**). Quantification of NLRP3 (**G**), GSDMD-NT (**H**) and Caspase-1 p20 (**I**) normalized to β-actin levels (*n* = 4). (**J**) Biaxial scatter plots showing the distribution and expression of Cybb and Trpm2 across all cell populations in the VBI group. (**K**) Immunofluorescence imaging of CD68 and GSDMD co-staining in left common carotid arteries from VBI and Sham rats (*n* = 6). (**L** and **M**) Representative images of hematoxylin and eosin-stained left common carotid arteries from shCtrl and shGSDMD rats (**L**). Ratio of intima to media (intima/media ratio) measured in hematoxylin and eosin staining images from each group (**M**) (*n* = 5). Data are presented as mean ± standard deviation (SD). Statistical significance was determined by one-way analysis of variance (ANOVA) with Tukey’s test. **P < 0.01, ***P < 0.001

We performed high-sensitivity transcriptomic sequencing and analysis on the left common carotid arteries of rats. Differential expressed gene (DEG) analysis revealed 2808 genes significantly upregulated, and 1178 genes significantly downregulated in vessels treated with balloon vascular injury (VBI) compared to control (Sham) vessels (Figure S1A). Notably, genes closely associated with pyroptosis, including *GSDMD*, *CASP1*, *IL1b*, *IL18*, *NLRP3*, *AIM2*, *NLRC4*, were upregulated in the VBI group compared to the control group (Figure S1B). Macrophage-related genes, such as *CD68*, were upregulated in the model group with vascular balloon injury (VBI) (Figure S1C). Gene expression analysis related to VSMC phenotypic switching indicated an upregulation of synthetic phenotype markers and a downregulation of contractile phenotype markers in the VBI group (Figure S1D). In the VBI group, inflammatory EC-related genes, including *ICAM1* and *VCAM1*, were upregulated (Figure S1E). The heatmap of these genes in the Sham and VBI groups displayed results consistent with the volcano plots (Fig. 1A; Figure S1F, G and H). Gene Ontology (GO) enrichment analysis indicated that terms related to the recruitment, migration, activation, and pyroptosis of monocytes/macrophages were enriched (Fig. 1B). Kyoto Encyclopedia of Genes and Genomes (KEGG) pathway enrichment analysis revealed that inflammation and pyroptosis-related pathways were altered following VBI, including the Chemokine signaling pathway, Leukocyte transendothelial migration, Calcium signaling pathway, NOD-like receptor signaling pathway, Toll-like receptor signaling pathway, and Cytokine-cytokine receptor interaction (Figure S1I). Additionally, we downloaded and reanalyzed the transcriptomic dataset (GSE179645) containing blood samples from patients with and without restenosis after endovascular interventions. The analysis revealed elevated expression levels of IL-1β, IL-18, HMGB1, and LDH in the blood of patients with restenosis (Figure S2A-E). Although distinct in certain aspects, the mechanisms underlying stenosis of vein grafts after coronary artery bypass grafting (CABG) share similarities with restenosis after endovascular interventions, including endothelial dysfunction, neointimal hyperplasia, monocyte infiltration, and inflammatory responses [46, 47]. Consequently, targeted analysis of the RNA-seq dataset (GSE241205), designed to assess transcriptomic differences between occluded vein grafts and healthy veins from human, revealed an upward trend in the expression levels of pyroptosis-related genes in occluded vein grafts (Figure S2F-O). These results indicate that pyroptosis is involved in the inflammation of VBI arteries.

To further explore the specific cell populations undergoing pyroptosis after VBI, single-cell RNA sequencing (scRNA-seq) was performed. We retained cells with mitochondrial gene content below 33% and analyzed a total of 18,077 cells from the left common carotid arteries of rats in the Sham and VBI groups. Using Uniform Manifold Approximation and Projection (UMAP), we identified VSMCs, ECs, fibroblasts, pericytes, Schwann cells, monocytic cells, neutrophils, B cells, and T cells (Fig. 1C and D). Each cell type exhibited distinct gene expression profiles (Figure S3A and B). Compared to the Sham group, a new population of monocytic cells emerged in the injured arteries (VBI group) (Fig. 1D). Further sub-clustering of the monocytic population revealed that the newly recruited monocytic cells in the VBI group were predominantly macrophages, accounting for approximately 92% of this population (Figure S3C). Interestingly, key genes involved in pyroptotic inflammation were predominantly highly expressed and specifically localized in the monocytic population following VBI (Fig. 1E), suggesting the potential activation of multiple inflammasomes. CYBB is involved in the production of reactive oxygen species (ROS), which triggers pyroptosis [48]. TRPM2 regulates calcium influx, activating inflammasomes [49]. It was observed that both *CYBB* and *TRPM2* were predominantly expressed in the monocytic population after VBI (Fig. 1J). These data indicate that pyroptosis in VBI arteries occurs primarily within the newly infiltrated macrophages.

The protein levels of pyroptosis-related biomarkers in the left common carotid arteries were measured using western blotting analysis (Fig. 1F, G, H and I). Compared to the control group, the protein levels of NLRP3, caspase-1 p20, and GSDMD-NT in the damaged arteries were significantly increased. We also examined the in-situ expression and cellular localization of GSDMD using immunofluorescence staining. Co-staining of GSDMD with the specific marker of macrophages (CD68), ECs (CD31), and SMCs (α-SMA) showed that GSDMD was localized in CD68^+^ macrophages (Fig. 1K; Figure S14). In addition, compared to the control group, the expression level of GSDMD and the number of CD68^+^ macrophages in the damaged arteries of VBI rats were significantly elevated (Fig. 1K), consistent with the transcriptomic analysis results. These findings suggest that following vascular intervention, multiple inflammasomes are activated, leading to GSDMD-mediated macrophage pyroptosis in the injured arteries.

Given the increased GSDMD-mediated pyroptosis of macrophages in the injured arteries following vascular intervention, we sought to determine whether knockdown of GSDMD, the final common effector downstream of multiple inflammasome activations, could attenuate the development of restenosis. To specifically knock down macrophage GSDMD in the injured vessels, we employed viral transduction using AAV-F4/80 carrying short hairpin RNA (shRNA) targeting GSDMD [50–52]. The selectivity of AAV-F4/80 was validated through local and systemic co-injection, resulting in specific and efficient transduction of macrophages in the injured vessels (Figure S15A). The transduction efficiency was assessed by Western blot, showing that the GSDMD protein level in macrophages from injured arteries of shGSDMD rats was decreased by approximately 50% compared with scrambled control shRNA rats (Figure S15B). Next, the restenosis was evaluated in injured rat arteries. shGSDMD rats showed significantly improved restenosis conditions, as assessed by reduced inflammation, enhanced re-endothelialization, and decreased intima/media ratio (Fig. 1L and M; Figure S15C, D, E, and F). In aggregate, these findings suggest that the increased GSDMD-mediated macrophage pyroptosis in injured vessels following endovascular intervention exacerbates inflammation, contributing to endothelial injury and neointimal hyperplasia.

### Characterization and precise endovascular delivery of disulfiram-coated balloon (DCB)

**Fig. 2:**
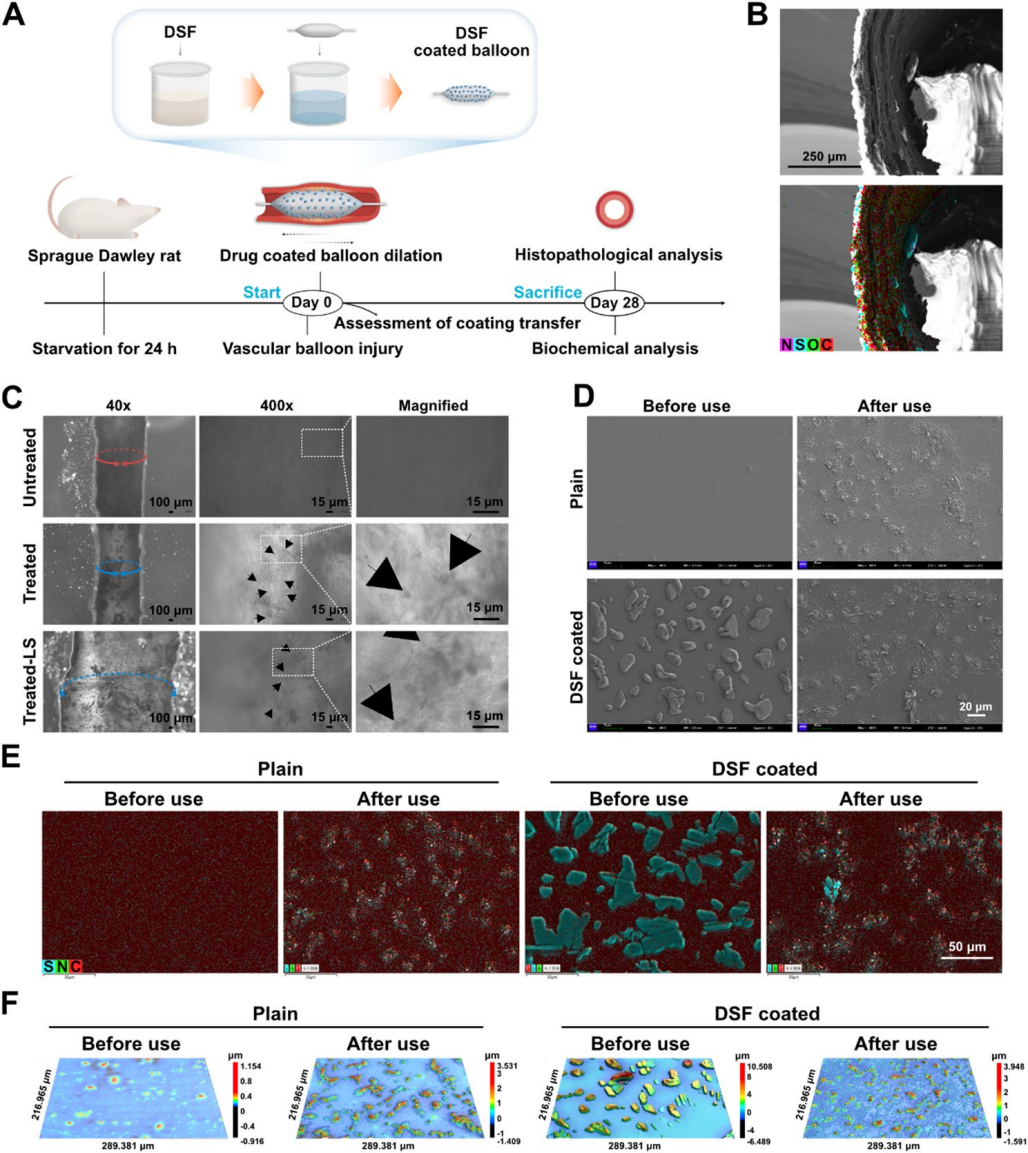
Characterization and precise endovascular delivery of disulfiram-coated balloon (DCB). (**A**) Schematic illustrating the fabrication process of disulfiram coated balloon and the establishment of the vascular balloon injury model with simultaneous treatment using different drug coated balloons. (**B**) Element mapping of the left common carotid artery treated with DCB. (**C**) In vivo coating transfer and distribution in the left common carotid arteries of rats. Phase-contrast imaging of the arteries with or without DCB treatment. LS: longitudinal section. Black arrows indicate DSF particles transferred from the balloon surface to the vessel wall. (**D**) Scanning electron microscopy (SEM) imaging of the surface morphology of plain balloons and DSF coated balloons before and after use. (**E**) Element mapping of the balloons before and after use. (**F**) 3D optical surface profilometry (OSP) images of balloon surfaces before and after use

Given the above findings point to the critical role for GSDMD-mediated macrophage pyroptosis in vascular restenosis. We next sought to develop an anti-restenosis therapeutic strategy by targeting macrophage pyroptosis. The aforementioned scRNA-seq results indicate the activation of multiple inflammasomes, including NLRP1, NLRP3, NLRC4, AIM2, and Pyrin inflammasomes, in the injured arteries following endovascular intervention. This suggests that therapies targeting any single inflammasome, such as NLRP3 inflammasome, may be insufficient due to compensatory activation of other inflammasomes. Additionally, although anti-IL-1β or anti-IL-18 biologics can partially neutralize pro-inflammatory cytokines, they cannot prevent the continuous release of these cytokines through pyroptosis. Disulfiram (DSF), the active ingredient of the FDA-approved drug Antabuse^®^, has established clinical safety and functions by inhibiting aldehyde dehydrogenase (ALDH), inducing an aversive flushing response to discourage alcohol consumption [41, 53]. Importantly, DSF is a potent inhibitor of pyroptosis by blocking GSDMD pore formation [32], suggesting that it may suppress inflammation induced by any inflammasome activation and mitigate the exacerbation of inflammation caused by the release of pro-inflammatory cytokines. Therefore, we designed a disulfiram-coated balloon (DCB) to directly target the injured vascular region and enhance the local drug concentration while minimizing systemic side effects, and evaluated the impact of targeting macrophage pyroptosis with DCB on restenosis after vascular intervention in vivo (Fig. 2A).

Incorporating polymers into the coating can enhance therapeutic agent loading; however, it may also induce inflammation and increase the difficulty of coating transfer into the vascular wall [54, 55]. Accordingly, given the lipophilic and hydrophobic properties of DSF [56], we examined the loading characteristics of a polymer-free DSF coating. DSF was passively adsorbed onto the balloon surface using the dip coating procedure [57], which is one of the industry-standard coating techniques (Fig. 2A; Figure S10). Brightfield imaging of the surface morphology of DCB provided preliminary validation of DSF loading on the balloon surface (Figure S11). Scanning electron microscopy (SEM) imaging showed that disulfiram particles were uniformly loaded and distributed on the balloon surface (Fig. 2D). The DSF coating exhibited a crystalline rather than amorphous structure, indicating enhanced potential for effective transfer into the target vascular wall [58–60]. Elemental mapping results further demonstrated the loading and distribution of DSF on the balloon surface (Fig. 2E). Consistently, 3D optical surface profilometry (OSP) imaging revealed the distribution of DSF particles on the balloon surface (Fig. 2F). The high-performance liquid chromatography (HPLC) analysis of the eluate from the coated balloon also confirmed the successful loading of DSF onto the balloon (Figure S12).

The capacity of the crystalline coating on DCB to transfer into the vascular wall through balloon dilation was subsequently assessed. To monitor the accumulation of DSF in the carotid artery wall after endovascular intervention, we utilized SEM, elemental mapping, and OSP to observe the balloon’s surface morphology and the deposition of DSF particles. (Fig. 2D, E and F). After VBI, the DSF particles on the surface of the coated balloon decreased, accompanied by some blood cells remaining on the surface. Additionally, to exclude the possibility of the coating drug being washed away by blood flow, ex vivo phase-contrast microscopy imaging was performed to directly observe the vessels treated with and without VBI. A noticeable accumulation of DSF particles, ranging in size the nanoscale to the microscale, was observed in the carotid artery wall following VBI (Fig. 2C). This range in DSF particle size is advantageous for enabling localized sustained release [58]. Cross-sectional imaging of the DCB-treated vessel using SEM, consistent with phase-contrast imaging results, revealed the accumulation of DSF particles in the damaged vessel wall (Fig. 2B; Figure S13). These data indicate that DCB possesses the excellent transfer capacity of the coating into the injured vessel wall via dilation.

### DCB inhibits GSDMD-mediated macrophage pyroptosis and associated inflammation in a rat carotid artery model

**Fig. 3:**
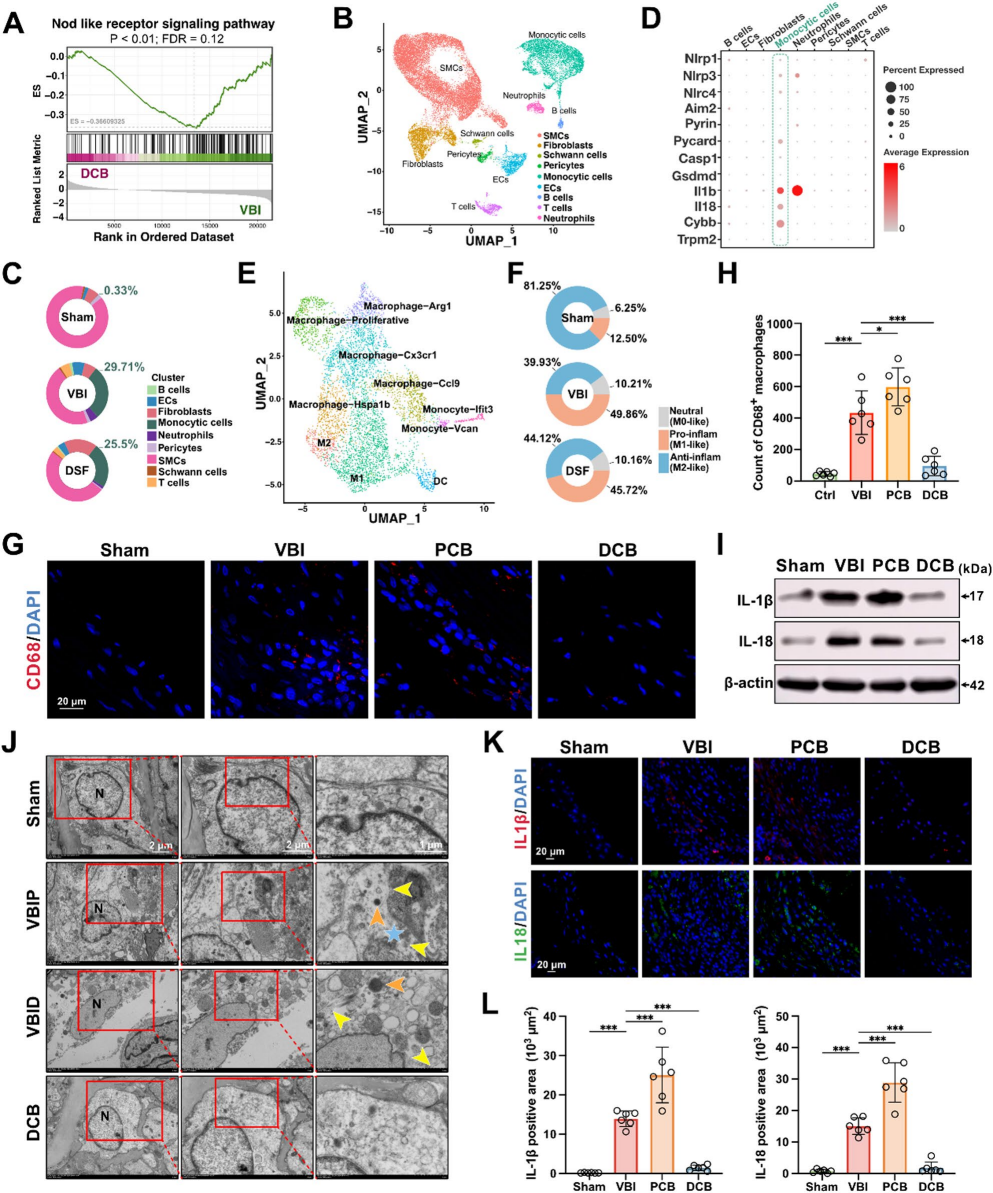
DCB inhibits GSDMD-mediated macrophage pyroptosis and associated inflammation in a rat carotid artery model. (**A**) The Gene Set Enrichment Analysis (GSEA) of differentially expressed genes between the DCB group and the VBI group. (**B**) Cells from the common carotid arteries of rats in the Sham group, VBI group and DCB group were analyzed using scRNA-Seq. Visualization of clustering of arterial cells using a UMAP plot. (**C**) Pie charts showing the proportion of each cell population in the Sham group, VBI group, and DCB group. (**D**) Dot plot showing the average scaled expression levels of key genes related to pyroptotic inflammation across different cell populations. (**E**) UMAP plot showing 10 distinct monocytic subpopulations. (**F**) Pie charts showing the proportion of anti-inflammatory (Anti-inflam), pro-inflammatory (Pro-inflam), and neutral cells within monocytic populations across the groups. (**G** and **H**) Representative immunofluorescence images of CD68 (red) stain taken from left common carotid arteries from control and VBI rats with or without PTX coated balloon (PCB) or DCB (**G**). DAPI stained in blue. Quantification of the numbers of CD68^+^ cells (**H**) (*n* = 6). (**I**) Western blotting analysis of left common carotid arteries from four indicated groups of rats at 4 weeks (*n* = 4). (**J**) Representative transmission electron microscopy (TEM) images of macrophages from the left common carotid arteries of rats in the control, VBI, or DCB groups. N: Nucleus. VBIP: Macrophage in the VBI group suspected of undergoing pyroptosis. VBID: Macrophage in the VBI group suspected of dying. Yellow arrows: Pore formation and discontinuity of the cell plasma membrane. Orange arrows: Lysosomes. Light blue asterisk: Cytoplasmic leakage. (*n* = 3). (**K** and **L**) Representative immunofluorescence images of IL-1β (red) and IL18 (green) staining taken from left common carotid arteries from four indicated groups of rats (**K**). Quantification of total IL‐1β and IL18 (**L**) positively staining area at 4 weeks (*n* = 6). Data are presented as mean ± standard deviation (SD). Statistical significance was determined by one-way analysis of variance (ANOVA) with Tukey’s test. ***P < 0.001

Next, it was evaluated whether DCB has inhibitory effects on pyroptosis and associated inflammation in the injured arteries of rats. Transcriptomic analysis between the DCB group and the VBI group showed that 1018 genes were significantly upregulated, and 1230 genes significantly downregulated in DCB-treated vessels compared to VBI-treated vessels (Figure S4A). Genes closely associated with pyroptosis were downregulated in the DCB group compared to the VBI group (Figure S4B). Macrophage-related genes, such as *CD68*, were downregulated in the DCB group (Figure S4C). Contractile phenotype markers of VSMCs were upregulated in the DCB group (Figure S4D). Inflammatory EC-related genes, including *ICAM1* and *VCAM1*, were downregulated in the DSF group (Figure S4E). The heatmap of these genes in the VBI and DCB groups displayed results consistent with the volcano plots (Figure S4F, G, H and I). GO enrichment analysis of DEGs between the DCB and VBI groups indicated terms related to cytoskeletal remodeling, cytokine regulation, inflammatory response, regulation of macrophage chemotaxis, and modulation of VSMC phenotype (Figure S4J). Consistently with GO enrichment analysis, KEGG pathway enrichment analysis of DEGs between the DCB and VBI groups revealed that DCB may inhibit VBI-induced changes in cytoskeletal organization, cytokine release, inflammatory response, and VSMC phenotype transformation (Figure S4K). Gene Set Enrichment Analysis (GSEA) indicated that the NOD-like receptor signaling pathway and other pyroptosis-associated inflammatory activation pathways were enriched in the VBI group compared to the DCB group (Fig. 3A; Figure S6 and S7). These findings suggest that DCB may exert protective effects on injured vessels by modulating pyroptosis-related inflammation.

To investigate the effects of DCB treatment on macrophages, scRNA-seq was performed on the left common carotid arteries of DCB-treated rats (Fig. 3B; Figure S5A). Key pyroptosis-related genes were primarily expressed in the monocytic population (Fig. 3D), and the mRNA levels of these genes showed a decrease in the DCB group compared to the VBI group (Figure S8 and S9). Arteries of DCB-treated rats displayed a reduction in the monocytic population compared to the VBI group (Fig. 3C), and the proportion of pro-inflammatory macrophages was also reduced in the DCB group compared to the VBI group (Fig. 3E, F;Figure S5B). Additionally, pseudotime analysis was performed on the monocytic cells. Dendritic cells (DCs) were positioned at the early stage of the developmental trajectory, while macrophages were located at the late stage (Figure S5C, D, E and F). The expression analysis of CD68, NLRP3, CASP1, and GSDMD during pseudotime revealed that *CD68*, *CASP1*, and *GSDMD* simultaneously increased in the early and mid-stages of pseudotime and then decreased concurrently in the late stage (Figure S5H, I, J and K). This suggests that macrophages may undergo pyroptosis in the late pseudotime stage, leading to a sudden drop in the expression of key genes involved in pyroptotic inflammation. *NLRP3* expression showed a slight decline in the early and mid-stages, but the existing *NLRP3* inflammasomes were sufficient to sustain the activation of downstream signaling [61, 62]. Additionally, *GSDMD* expression was positively correlated with mitochondrial gene content (Figure S5G), indicating that cells undergoing pyroptosis with high GSDMD expression may have been filtered out in earlier analyses. This could explain why GSDMD was not as specifically concentrated in the monocytic population compared to other key genes involved in pyroptotic inflammation (Fig. 1E). The changes in the expression of key pyroptosis-related genes in the monocytic population along the pseudotime trajectory further suggest macrophage pyroptosis in the injured vessels. These findings indicate that DCB inhibits macrophage pyroptosis and associated inflammation in injured arteries.

Macrophage recruitment in the injured carotid artery wall following VBI was in situ assessed by CD68 immunostaining (Fig. 3G). Compared to rats treated with the plain balloon for the VBI procedure, rats treated with PTX coated balloon (PCB) exhibited a significant increase in macrophage infiltration in the carotid artery wall, while DCB treatment significantly reduced macrophage recruitment, consistent with the scRNA-seq analysis of DCB-treated rat arteries (Fig. 3H). The ultrastructure of the common carotid artery was further examined using transmission electron microscopy (TEM). Macrophages in the VBI group showed pore formation and discontinuity of the cell plasma membrane, ballooning protrusions, and cellular fragments, which are signs of macrophage pyroptosis [63]. In contrast, macrophages in the DCB group largely preserved normal ultrastructural features, with intact plasma membranes, well-defined cytoplasmic boundaries, and organized intracellular structures, indicating effective suppression of macrophage pyroptosis (Fig. 3J). To further evaluate the effects on pyroptosis-related inflammation in the injured vessels, key pro-inflammatory factors associated with pyroptosis were analyzed by western blotting of the left common carotid arteries. The carotid arteries of VBI rats exhibited elevated expression levels of IL-1β and IL-18, while DCB treatment significantly reduced these cytokines expression. Conversely, compared to VBI rats, the carotid arteries of rats treated with PCB showed increased IL-1β and IL-18 expression (Fig. 3I; Figure S16). Consistent with the western blotting results, immunofluorescence staining of IL-1β and IL-18 in the left common carotid arteries showed that DCB treatment markedly reduced IL-1β and IL-18 positive stained area (Fig. 3K and L). Taken together, these results indicate that DCB effectively suppresses GSDMD-mediated macrophage pyroptosis and associated inflammation in the injured vessels.

### DCB promotes functional re-endothelialization in a rat carotid artery model

**Fig. 4:**
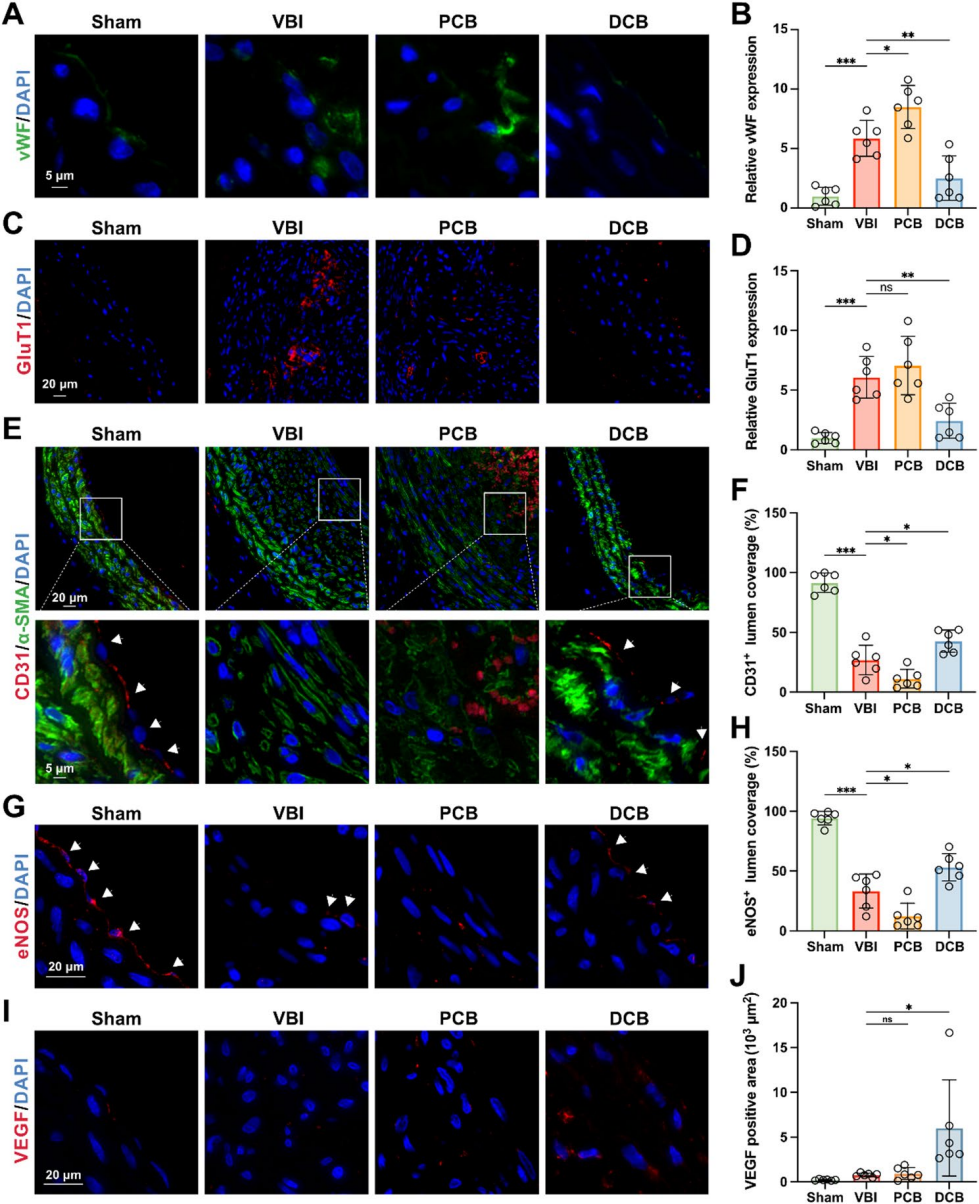
DCB promotes functional re-endothelialization in a rat carotid artery model. (**A** and **B**) Representative immunofluorescence images of vWF (green) in the common carotid artery (**A**). Quantification of the relative vWF (**B**) expression in the common carotid artery (*n* = 6). (**C** and **D**) Representative immunofluorescence images of GluT1 (red) in the common carotid artery (**C**). Quantification of the relative GluT1 (**D**) expression in the common carotid artery (*n* = 6). (**E** and **F**) Representative immunofluorescence images of CD31 (red) and α-SMA (green) staining taken from left common carotid arteries from four indicated groups of rats at 4 weeks(**E**). Quantification of CD31 (**F**) staining (*n* = 6). (**G** and **H**) Representative immunofluorescence images of eNOS (red) staining taken from left common carotid arteries from four indicated groups of rats (G). Quantification of eNOS (H) staining (*n* = 6). (**I** and **J**) Representative immunofluorescence images of VEGF (red) staining taken from left common carotid arteries from four indicated groups of rats(**I**). Quantification of VEGF (**J**) staining (*n* = 6). Data are presented as mean ± standard deviation (SD). Statistical significance was determined by one-way ANOVA with Tukey’s test. *P < 0.05, **P < 0.01, ***P < 0.001. ns, no significant difference

The mechanical damage from the VBI procedure, such as dilation and friction, disrupts the integrity and function of ECs, creating an opportunity for the excessive proliferation and migration of SMCs, ultimately leading to restenosis [14]. Therefore, we next investigated the effects of DCB on the endothelium of injured rat arteries. DCB reduced the expression of von Willebrand Factor (vWF) in the intima (Fig. 4A and B), and the downregulation of vWF can promote the proliferation of ECs [64]. Glucose transporter-1 (GluT1) is used to assess immature intimal neovascularization and is closely associated with late-stage complications [65]. The intima in the DCB group showed decreased GluT1 expression (Fig. 4C and D). To evaluate the impact of different balloon treatments on functional re-endothelialization following VBI, we conducted immunostaining for endothelial coverage (CD31) and function (eNOS) in the vessels. The carotid arteries treated with a plain balloon exhibited incomplete and patchy CD31^+^ and eNOS^+^ endothelial coverage, whereas those treated with DCB demonstrated significantly increased CD31^+^ and eNOS^+^ endothelial coverage (Fig. 4E, F, G and H). A complete and functional endothelium can prevent excessive migration and proliferation of underlying SMCs [14]. Arteries treated with the DCB exhibited significantly reduced positive staining area of the SMCs specific marker α-SMA (Fig. 4E; Figure S17). Vascular endothelial growth factor (VEGF) promotes endothelial growth [66]. Immunofluorescence staining for VEGF showed that DCB treatment significantly increased VEGF positive staining area, further confirming the effectiveness of DCB on endothelial cell recovery (Fig. 4I and J). Conversely, arteries treated with PCB showed significant inhibition of endothelial coverage (CD31) and function (eNOS) (Fig. 4E, F, G and H). Taken together, these data indicate that DCB treatment promotes functional re-endothelialization.

### DCB suppresses neointimal hyperplasia in rat carotid and Iliac artery models

**Fig. 5:**
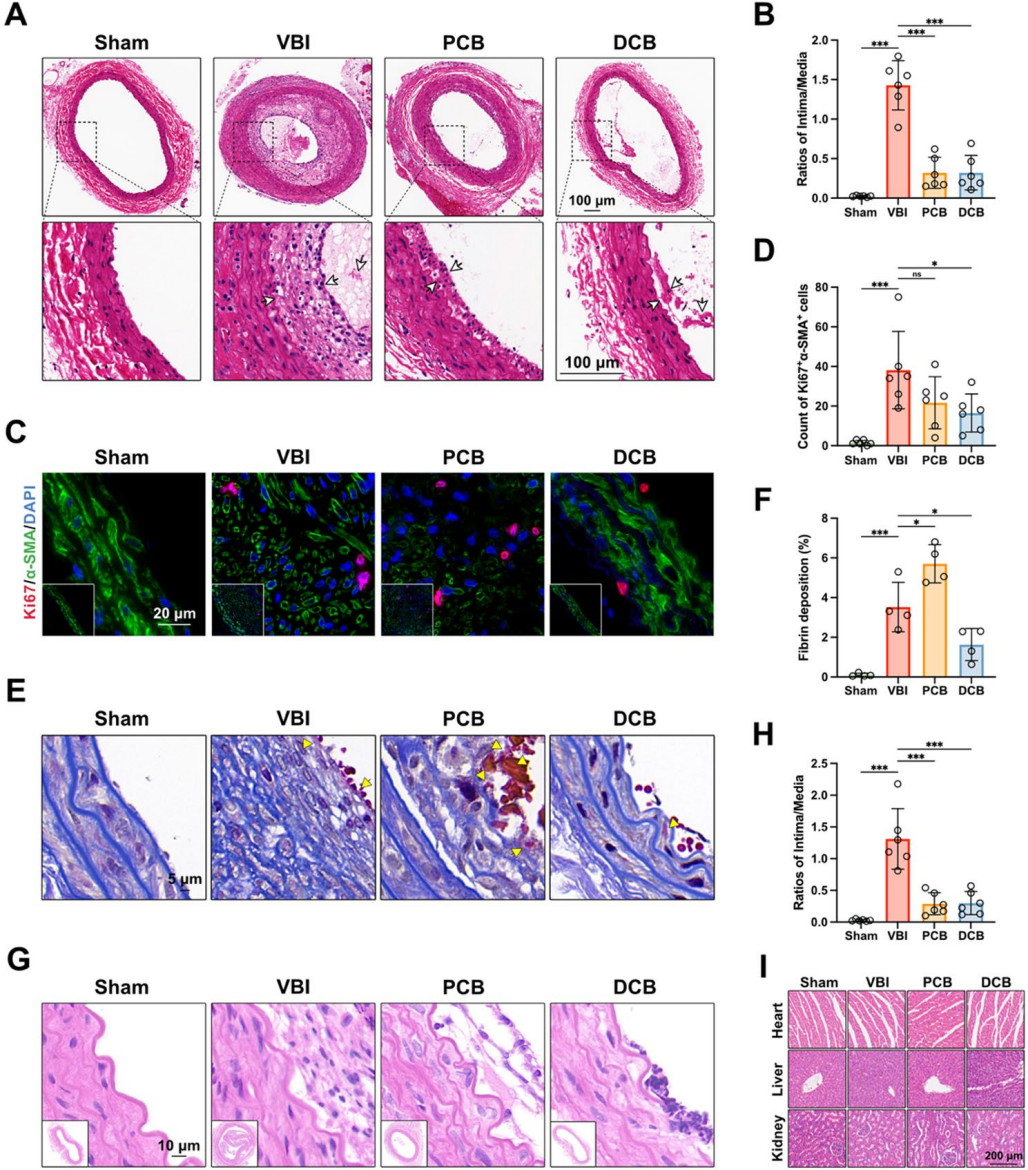
DCB suppresses neointimal hyperplasia in rat carotid and iliac artery models. (**A** and **B**) Representative images of hematoxylin and eosin (H&E)-stained left common carotid arteries from four indicated groups of rats at 4 weeks showing neointimal hyperplasia (**A**). Ratio of intima area to media area (intima/media ratio) measured in H&E staining images from each group (**B**) (*n* = 6). (**C** and **D**) Representative immunofluorescence images of α-SMA (green) and Ki67 (red) staining taken from left common carotid arteries from four indicated groups of rats (**C**). Quantitative analysis of the numbers of α-SMA^+^ Ki67^+^ cells (**D**) (*n* = 6). (**E** and **F**) Representative images of Martius Scarlet Blue (MSB) stained left common carotid arteries from four indicated groups of rats (**G**). Incarnadine: fresh fibrin (demonstrated with yellow arrows); bluish violet: stale fibrin; sapphire: collagen fibers; gray: platelet trabeculae; bluish brown: nucleus; yellow: RBCs. Quantification of fresh fibrin (incarnadine) deposition (H) within left common carotid arteries, expressed as a percentage of the positively stained area relative to the total lumen area (*n* = 6). (**G** and **H**) Representative images of H&E-stained common iliac arteries from four indicated groups of rats at 4 weeks showing neointimal hyperplasia (**I**). Ratio of intima to media measured in H&E staining images from each group (**J**) (*n* = 6). (**I**) Representative H&E-stained images of major organs, including the heart, liver, and kidneys, to assess the biosafety of various treatments (*n* = 6). Data are presented as mean ± standard deviation (SD). Statistical significance was determined by one-way ANOVA with Tukey’s test. *P < 0.05, **P < 0.01, ***P < 0.001. ns, no significant difference

To assess the ultimate functional outcomes of the novel DCB treatment for vascular restenosis, the left common carotid arteries were stained with hematoxylin and eosin (H&E) to measure the development of neointimal hyperplasia (NIH), which provides direct evidence of the DCB’s therapeutic effects on vascular restenosis. Compared to control group, VBI rats exhibited a renewed lumen loss due to the contraction of the expanded vessels after removing the balloon (Fig. 5A), consistent with the findings of Rozenman Y [67], as also shown in Fig. 2C. The arteries of VBI rats showed obvious neointimal hyperplasia at 28 days, however, DCB treatment reduced neointimal formation (Fig. 5A). Further morphological analysis revealed that, vessels treated with DCB exhibited a reduced intima to media ratio, indicating an attenuation of restenosis severity (Fig. 5B).

SMCs switch from the contractile to the synthetic phenotype in response to vascular injury following VBI, which manifests as excessive proliferation and migration, contributing to restenosis [16]. Therefore, we examined the immunofluorescent co-staining of the SMCs specific marker α-SMA with common proliferation marker Ki67 (Fig. 5C and D). The results showed that the left common carotid arteries of rats subjected to the VBI procedure with the plain balloon displayed an increase in α-SMA^+^Ki67^+^ cells, whereas DCB treatment significantly reduced the number of α-SMA^+^Ki67^+^ cells. Commercial drug coated devices have reported high rates of late-stage thrombosis in long-term clinical studies [6, 12]. To evaluate the effects of different balloons on potential thrombosis, we analyzed fibrin deposition in the vessel wall using Martius Scarlet Blue staining (Fig. 5E and F; Figure S18). The VBI procedure with a plain balloon increased fibrin deposition in the injured arteries and PCB further exacerbated fibrin deposition. Interestingly, the left common carotid arteries of rats treated with the DCB showed significantly reduced fibrin deposition. These results are consistent with the GSEA, where the complement and coagulation cascades pathways were enriched in the VBI group compared to the DCB group (Figure S6). The iliac artery is also a common site for stenosis in clinical practice [68]. Thus, a common iliac artery balloon injury model was further selected to evaluate the applicability and efficacy of the coated balloon in different vascular sites. The DCB group demonstrated reduced neointimal hyperplasia in the common iliac artery (Fig. 5G and H). Additionally, H&E staining of major organs, including the heart, liver, spleen, lungs, and kidneys, indicated that the DCB exhibited good biosafety (Fig. 5I; Figure S20). Taken together, these data demonstrate that DCB treatment can more effectively suppress long-term restenosis by inhibiting pyroptosis and associated inflammation in injured vessels after endovascular intervention.

### DSF curbs macrophage pyroptosis and associated inflammation

**Fig. 6:**
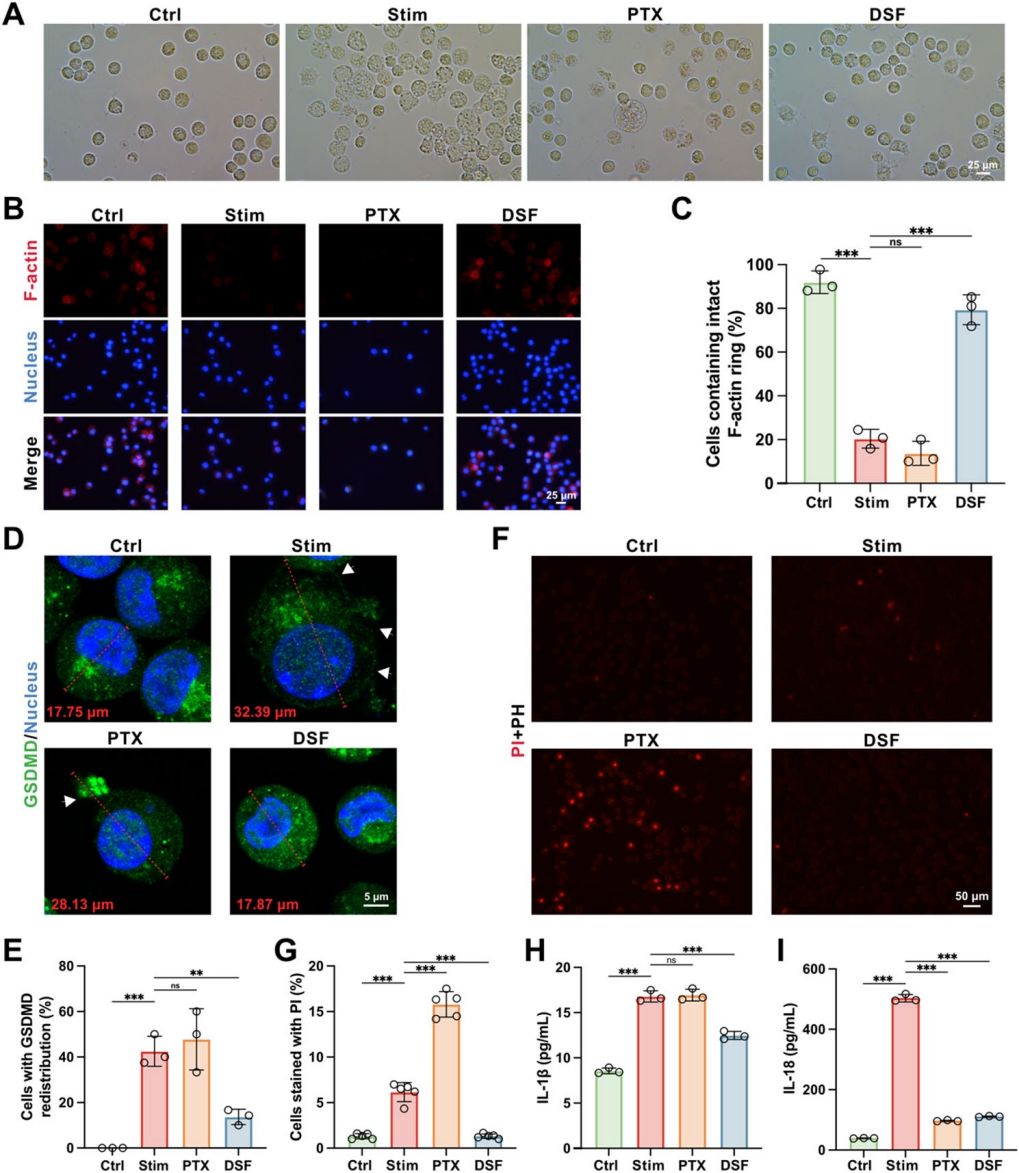
DSF curbs macrophage pyroptosis and associated inflammation. Macrophages were primed with LPS (1 µg/mL), pretreated or not with DSF (20 µM) or PTX (20 µM) for 1.5 h and stimulated with ATP (2.5 mM). Stim refers to the group treated with only LPS and ATP. (**A**) Representative phase-contrast images of macrophages showing the morphological membrane ballooning characteristic of pyroptotic cells (*n* = 6). (**B** and **C**) Representative immunofluorescence images of F-actin (red) staining (**B**). Quantification of F-actin (**C**) staining (*n* = 3). (**D** and **E**) Representative immunofluorescence images of GSDMD (green) staining. Quantification of the numbers of cells with GSDMD redistribution to the plasma membrane and pyroptotic bubbles (*n* = 3). White arrowheads indicate GSDMD staining of pyroptotic bubbles. Red lines denote the dimension of representative individual cell. (**F** and **G**) Representative fluorescence imaging of cells stained with propidium iodide (PI) (red) along with corresponding phase-contrast imaging (**F**). Quantification of the numbers of cells taking up PI (**G**) (*n* = 5). (**H** and **I**) Levels of the cytokines IL-1β (**H**) and IL-18 (**H**) in culture supernatants (*n* = 3). Data are presented as mean ± standard deviation (SD). Statistical significance was determined by one-way ANOVA with Tukey’s test. **P < 0.01, ***P < 0.001. ns, no significant difference

Although the inhibitory effect of DSF on pyroptosis is evident, it cannot exclude the possibility that DSF may also exert direct effects on two other key vascular cell types through alternative pathways. To address this issue, we established a pyroptotic macrophage model with inflammasome activation through treatment with lipopolysaccharide (LPS) and adenosine 5’-triphosphate disodium salt hydrate (ATP). When LPS-primed macrophages were treated with ATP in the absence of DSF, increasing pyroptotic membrane ballooning was morphologically observed (Fig. 6A). However, DSF, added at 1.5 h before ATP, effectively inhibited pyroptotic membrane ballooning. Consistent with the result of morphological observation, fluorescent staining of F-actin revealed that DSF rescued stimulated macrophages from pyroptosis, while PTX treatment exacerbated cell death (Fig. 6B and C). Intact plasma membrane prevents propidium iodide (PI) from staining the nucleus. PI staining showed that stimulated macrophages took up PI red, whereas DSF treatment inhibited nuclear staining (Fig. 6F and G). Immunofluorescent staining for GSDMD revealed its presence in both cytosol and plasma membrane of stimulated macrophages, forming characteristic pyroptotic bubbles. Interestingly, DSF treatment inhibited GSDMD membrane staining, the formation of pyroptotic bubbles, and membrane ballooning (Fig. 6D and E). Assessment of IL-1β and IL-18 levels in the culture supernatant showed significantly elevated levels of these key cytokines released by stimulated macrophages, which were markedly reduced by DSF treatment (Fig. 6H and I). Collectively, these results indicate that DSF has a potent inhibitory effect on GSDMD-mediated pyroptosis and associated inflammation in macrophages.

### DSF does not impair endothelial cell function

**Fig. 7:**
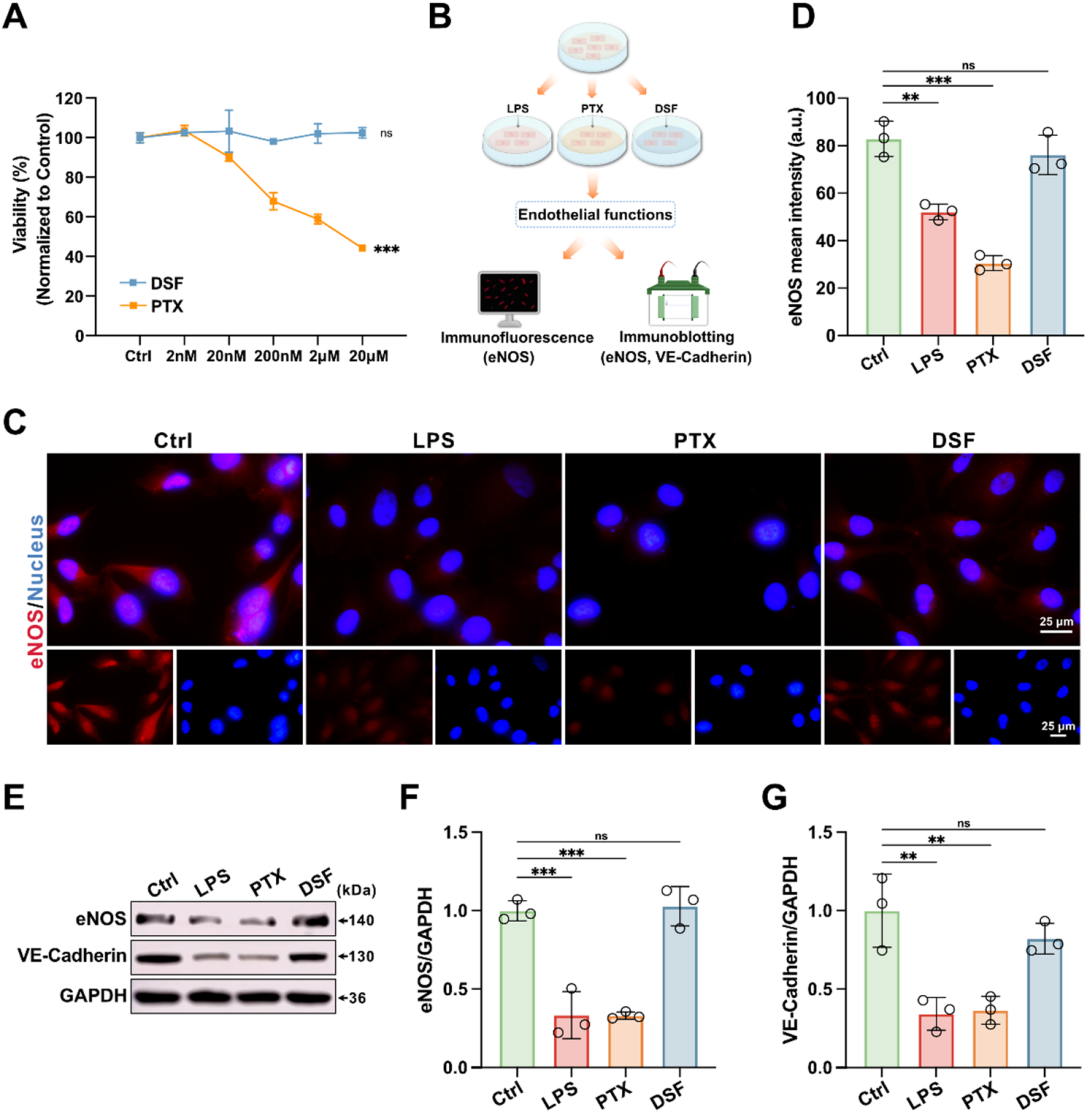
DSF does not impair endothelial cell function. (**A**) Viability of endothelial cells (ECs) treated with different doses of DSF or PTX for 12 h (*n* = 3). (**B**) Schematic illustration of the evaluation of endothelial cell function in vitro. ECs were treated with LPS and ATP or PTX or DSF. (**C** and **D**) Representative immunofluorescence images of eNOS (red) staining (**C**). Quantification of eNOS (**D**) staining (*n* = 3). (**E**-**G**) Western blotting analysis of ECs (**E**). Quantification of eNOS (**F**) and VE-Cadherin (**G**) normalized to GAPDH levels (*n* = 3). Data are presented as mean ± standard deviation (SD). Statistical significance was determined by one-way ANOVA with Tukey’s test. **P < 0.01, ***P < 0.001. ns, no significant difference

Next, we treated the key cell types individually with DSF to evaluate its direct effects on these cells. Non-specific cytotoxicity of the conventional drug coated balloons with anti-proliferative agents can impair functional endothelial healing, which hinders the long-term success of revascularization in clinical settings [6]. Therefore, the viability of ECs subjected to different treatments was assessed using the cell counting kit-8 (CCK-8) assay (Fig. 7A). The results showed that treatment with 20 µM DSF did not affect endothelial cell viability, whereas the corresponding dose of PTX significantly reduced cell viability. To evaluate the impact of different treatments on endothelial cell function, the levels of endothelial nitric oxide synthase (eNOS) were measured using immunofluorescent staining. As expected, PTX significantly reduced eNOS levels, whereas endothelial cells treated with DSF showed no significant difference in eNOS expression compared to the control group (Fig. 7B, C and D). Furthermore, we analyzed the levels of vascular endothelial cadherin (VE-cadherin) and eNOS in endothelial cells by western blotting to assess endothelial integrity and regulatory function (Fig. 7B, E, F and G). Consistent with the results of immunofluorescent staining, PTX treatment led to a reduction in VE-cadherin and eNOS levels, while DSF treatment did not result in significant changes in expression. Additionally, viability assays for SMCs and macrophages indicated that DSF treatment did not affect cell viability, whereas PTX treatment caused a marked decrease in viability for both cell types (Figure S19). Collectively, these findings suggest that DSF does not exert significant adverse effects on key vascular cell types.

### DSF regulates the migration and proliferation of endothelial cells and smooth muscle cells by inhibiting macrophage pyroptosis

**Fig. 8:**
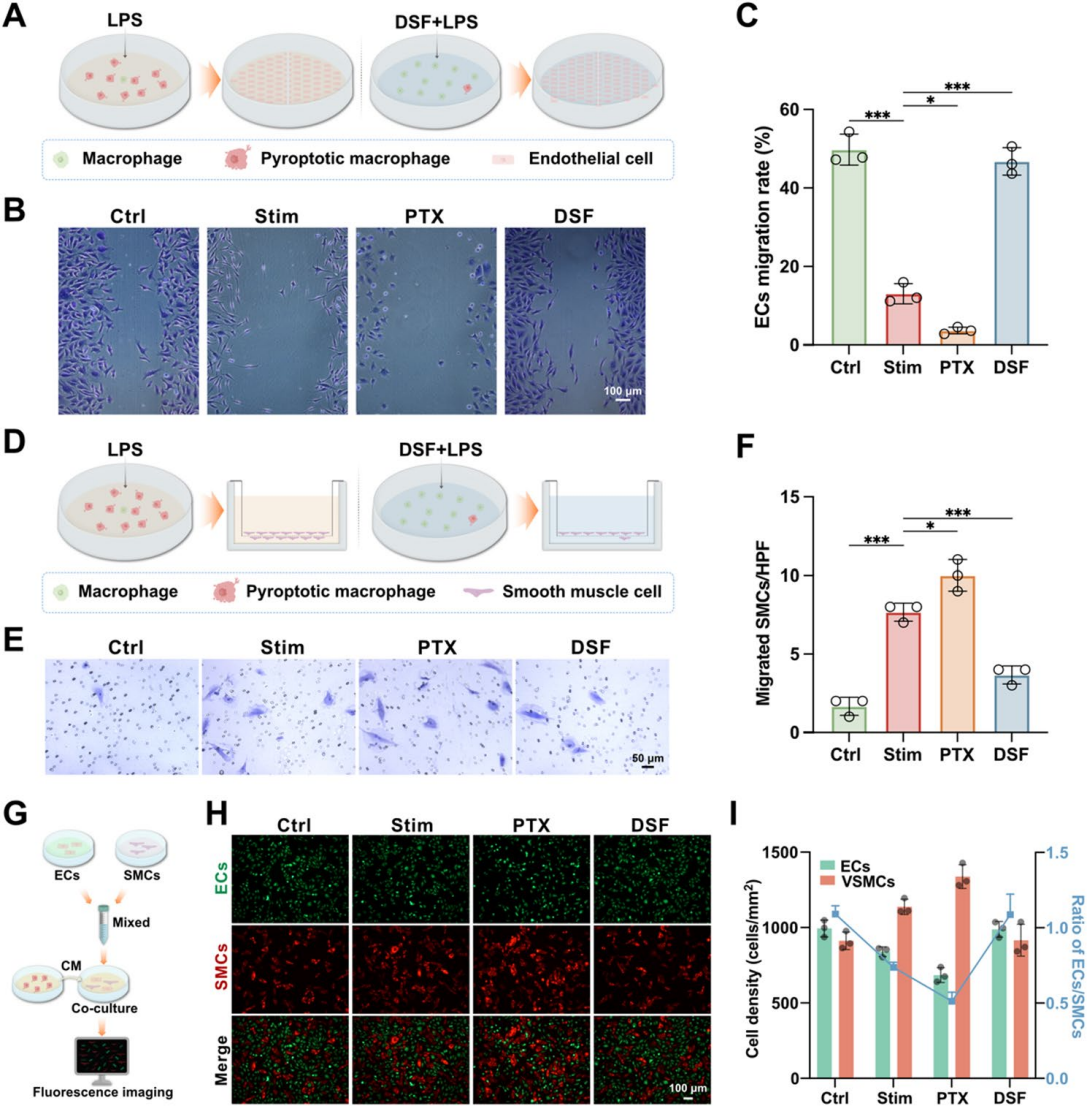
DSF regulates the migration and proliferation of endothelial cells and smooth muscle cells by inhibiting macrophage pyroptosis. (**A**-**C**) Schematic representation of wound healing assay of ECs after 24 h of co-culture (**A**). Representative images of cells migration (**B**). Quantification of cells migration rate among indicated groups (**C**). (**D**-**F**) Schematic representation of the transwell migration assay of SMCs after 24 h of co-culture (**D**). Representative images of smooth muscle cells stained with crystal violet (**E**). Quantification of the numbers of the migrated smooth muscle cells among indicated groups (**F**). (**G**-**I**) Schematic representation of the co-culture evaluation involving conditioned medium with endothelial and smooth muscle cells (**G**). Immunofluorescence imaging of co-culture systems among indicated groups, with ECs labeled in green and SMCs labeled in red (**H**). Quantification of cell density and the ratio of ECs to SMCs in co-culture systems (**I**). Data are presented as mean ± standard deviation (SD). Statistical significance was determined by one-way ANOVA with Tukey’s test. *P < 0.05, ***P < 0.001

In vivo results indicate that DCB treatment promotes re-endothelialization of injured vessels and inhibits smooth muscle cell proliferation and migration. Previous in vitro results have demonstrated that DSF does not directly affect the viability of ECs or VSMCs. To further investigate whether effects of DSF on the two major cell types (ECs and VSMCs) involved in vascular restenosis were partially mediated through the regulation of macrophage pyroptosis and its associated inflammation, we conducted in vitro experiments using conditioned medium (CM) from differently treated macrophages. Wound healing assay revealed that conditioned medium from stimulated macrophages significantly inhibited endothelial cell migration and proliferation. In contrast, conditioned medium from DSF-treated macrophages improved endothelial cell migration and proliferation (Fig. 8A, B and C). Transwell migration assay showed that conditioned medium from stimulated macrophages greatly enhanced SMC migration, which was reversed by the addition of DSF (Fig. 8D, E and F). To further simulate in vivo cellular interactions, ECs and SMCs were co-cultured, and conditioned medium from differently treated macrophages was then introduced into the co-culture system. Using different live-cell tracers to distinctly display the growth of ECs and SMCs, results showed that conditioned medium from macrophages treated with DSF increased the ECs/SMCs ratio (Fig. 8G, H and I). These findings suggest that DSF inhibits macrophage pyroptosis and its associated inflammation, thereby indirectly and selectively regulating the migration and proliferation of ECs and SMCs.

## Methods

### Materials and reagents

Disulfiram (DSF), paclitaxel (PTX) and phorbol 12-myristate 13-acetate (PMA) were from MedChemExpress. Lipopolysaccharide (LPS), Adenosine 5’-triphosphate disodium salt hydrate (ATP), Dimethyl sulfoxide (DMSO) and propidium iodide (PI) were from Sigma-Aldrich. ActinRed 555 Reagent was from ThermoFisher Scientific.

### Construction and characterization of the DSF coating

The balloons were disinfected in 75% ethanol and irradiated under ultraviolet (UV) light for 30 min. Drug loading was achieved by submerging the balloons in the high-concentration solution of DSF or PTX (5 mg/mL in ethanol) and incubating them overnight at 4 °C to ensure complete drug saturation [69]. The dried, coated balloons were consistently maintained at 4 °C, and then this procedure was repeated thrice with the samples dipped in the solution for 15 s. Coating characterization was performed using the inverted microscope (Leica Microsystems), scanning electron microscopy (SEM, Zeiss), and optical surface profilometry (OSP, Mahr).

### Determination of drug loading on DSF coated balloon

The deflated balloons were placed separately into brown bottles, and an appropriate amount of ethanol solution was added for extraction. The brown bottles were then placed into an ultrasonic water bath and treated for 15 min, followed by 1 min of vortex mixing. All collected solutions were analyzed using high-performance liquid chromatography (HPLC) to determine the amount of DSF released from the coated balloons.

### Balloon dilation in vivo

Animal experiments in this study were approved by the Animal Ethics Committee of Shanghai General Hospital, Shanghai Jiao Tong University School of Medicine (Grant No. 2024AW002). Male Sprague-Dawley (SD) rats (8 weeks old, 250–300 g) were purchased from the Experimental Animal Center of Shanghai General Hospital (Shanghai, China) and randomly assigned to control, VBI, PCB, and DCB groups. Rats in VBI group underwent vascular balloon injury (VBI) procedures [16, 70–75]. Briefly, after anesthesia and sterilization, a midline neck incision was made to expose the left common carotid artery. The balloon catheter was introduced into the left common carotid artery and inflated to 2 atm pressure, then withdrawn. This process was repeated three times. Rats in PCB group were treated with PCB to dilate the left common carotid artery. Similarly, rats in DCB group were treated with DCB to dilate the left common carotid artery. Control group rats underwent the same surgical procedure but without catheter insertion. The detailed procedure for the rat common iliac artery balloon injury model has been previously described [76, 77].

To evaluate the drug transfer from the coated balloons to the injured vessels, the treated arteries and balloons were immediately dissected after procedure and monitored using the inverted microscope, SEM, and OSP. Subsequently, the rats were sacrificed at 4 weeks post-surgery and the left common carotid arteries were collected for histopathological analysis, including H&E staining, immunofluorescence staining, and Martius Scarlet Blue (MSB) staining. Furthermore, immunoblot analysis of the common carotid arteries was performed 4 weeks post-surgery.

### Adeno-associated virus (AAV) infection in vivo

AAV9-F4/80-mCherry-5′miR-30a-shRNA (GSDMD)-3′miR-30a-WPREs (shGSDMD) or AAV9-F4/80-mCherry-5′miR-30a-shRNA (scramble)-3′miR-30a-WPREs (shCtrl) were constructed and packaged by HANBIO (Shanghai, China) [50, 52, 78–80]. For all constructs, titers were in the range of 10^12^-10^13^ viral genomes/ml. With the proximal and distal segments of the carotid artery temporarily clamped, 20 µl of undiluted virus was injected into the injured left common carotid artery. After a 30-minute incubation period, blood flow was restored, and the cervical incision was closed in layers [51, 81, 82]. One week later, a second AAV injection was administered, combining a 20 µl local injection into the carotid artery with a 60 µl systemic injection via the tail vein.

### RNA-seq analysis

Total RNA was extracted from the rat samples, and sequencing libraries were prepared. The constructed libraries were subjected to paired-end sequencing (PE150) on the Illumina NovaSeq™ 6000, following standard protocols. Data analysis was performed online for free on the LC-Bio Technologies cloud platform (www.lc-bio.com).

### Single-cell RNA sequencing and analysis

After harvesting the rat common carotid arteries, the tissues were rinsed with pre-chilled PBS and preserved in MACS Tissue Storage Solution (Miltenyi Biotec). The minced tissues were fully digested with Tissue Dissociation Solution and then filtered through a sterile cell strainer. After centrifugation, the supernatant was removed, and the cell pellet was resuspended in PBS to obtain a single-cell suspension. Cell viability was assessed using Calcein AM (Thermo Fisher Scientific) and Draq7 (BD Biosciences). Single-cell transcriptome data were captured using the BD Rhapsody system. Whole transcriptome libraries were prepared using the BD Rhapsody single-cell whole transcriptome amplification (WTA) workflow. Sequencing was performed on an Illumina sequencer with 150-base-pair paired-end reads. scRNA-seq data analysis was conducted using the NovelBrain cloud analysis platform (NovelBio Bio-Pharm Technology, sc.novelbrain.com).

Normalization was performed using the Seurat package (version 3.1.4). Principal component analysis (PCA) was conducted based on the top 2000 highly variable genes, and the first 10 principal components were used to construct tSNE and UMAP plots. Unsupervised cell clustering was obtained using a graph-based clustering method (resolution = 0.8), and marker genes were identified through the Wilcoxon rank-sum test. KEGG pathway analysis and GO analysis were employed to identify significant pathways and biological relevance of differentially expressed genes, using Fisher’s exact test with FDR correction for p-values. Single-cell trajectory analysis was performed using Monocle2 (cole-trapnell-lab.github.io). To systematically analyze intercellular communication, CellPhoneDB and CellChat were utilized to infer cell-cell interactions based on ligand-receptor pairs.

### H&E staining

The common carotid arteries of rats were harvested 4 weeks post-injury and fixed overnight at room temperature in 4% paraformaldehyde (PFA). The samples were dehydrated in ethanol, embedded in paraffin, and transversely sectioned into 5 μm slices to display the complete vascular ring. The sections were deparaffinized, rehydrated, and stained with Hematoxylin and Eosin. Images of the stained sections were captured using a microscope (Leica Microsystems) and analyzed with ImageJ software (National Institutes of Health). The measurements included neointimal area, medial area, intima area to media area ratio.

### Martius Scarlet blue staining

According to the manufacturer’s protocol, the modified Martius Scarlet Blue kit (Solarbio) was used to evaluate fibrin deposition in the carotid arteries. The amount of positive fibrin staining within the neointima was quantified using the Color Threshold and Analyze Particles in ImageJ, and the fibrin deposition was expressed as a percentage of the total lumen area.

### Cell culture and treatments

THP-1, HUVECs, and SMCs were all obtained from the American Type Culture Collection (ATCC) and maintained in a humidified air at 37 °C with 5% CO_2_. THP-1 were cultured in RPMI‐1640 medium, supplemented with 10% Fetal Bovine Serum (FBS), 100 U/mL penicillin and 100 µg/mL streptomycin, SMCs in smooth muscle cell growth medium (Merck), and HUVECs in endothelial cell growth medium (Merck). The cells were verified to be free of mycoplasma contamination. Generally, THP-1 cells were first cultured with PMA (50 nM) for 36 h to differentiate into macrophages. Subsequently, the macrophages were primed with LPS (1 µg/mL) for 3 h, followed by treatment with ATP (2.5 mM).

### Measurement of cytokines

According to the manufacturer’s instructions, the concentrations of IL-1β and IL-18 in the culture supernatants were measured using enzyme-linked immunosorbent assay (ELISA) kits (R&D Systems).

### Immunofluorescence staining

For the immunofluorescence analysis of the dried cross-sections of the common carotid arteries, the sections were stained with primary antibodies against CD68 (BioRad, MCA341GA), α-SMA (Abcam, ab7817), CD31 (Santa Cruz Biotechnology, sc-20071; Proteintech, 28083-1-AP), GSDMD (Proteintech, 20770-1-AP), IL-1β (Abcam, ab283818), IL-18 (Proteintech, 10663-1-AP), vWF (Proteintech, 66682-1-Ig), GluT1 (Proteintech, 21829-1-AP), eNOS (Abcam, ab300071), VEGF (Abclonal, A0280), and Ki67 (Abcam, ab16667). Detection of the primary antibodies was performed using fluorescent-conjugated secondary antibodies (Abcam). DAPI (Thermo Fisher Scientific) was used to stain the nuclei for 3 min. Fluorescence microscopy (Leica Microsystems) was employed to image the stained samples, focusing on the coverage of CD31 and eNOS positive cells and the area of positively stained cells for CD68, GSDMD, IL-1β, IL-18, α-SMA, Ki67, and VEGF. Each group consisted of 6 samples, and the immunofluorescence staining images were processed using ImageJ software for analysis.

For the immunofluorescence staining of cultured cells, the cells were fixed with 4% PFA and permeabilized with 0.1% Triton-X. Subsequently, the cells were blocked with 5% BSA diluted in PBST for 60 min. After blocking, the cells were incubated with primary antibodies against eNOS (Cell Signaling Technology, 32027 S) and GSDMD (Proteintech, 20770-1-AP) at 4 °C for 12 h. Following primary antibody incubation, the cells were washed with PBST and then incubated with secondary antibodies (Abcam, ab150078, ab150077) at room temperature for 1 h. Nuclei were stained with DAPI. The cells were visualized and imaged using an inverted fluorescence microscope. ImageJ software was used to measure the average fluorescence intensity of eNOS staining.

### Cell viability assay

According to the manufacturer’s instructions, cell viability was assessed using the Cell Counting Kit-8 (CCK-8) (Dojindo). Treated macrophages, endothelial cells, and smooth muscle cells were respectively seeded into 96-well plates and incubated at 37 °C until adherence. The optical absorbance density (OD) at 450 nm was measured using a microplate reader (Thermo Fisher Scientific). Untreated cells were used as the control group. The average OD values were normalized to the OD values of the control group to determine the cell viability percentage.

### Immunoblot analysis

To prepare tissue or cell extracts, samples were lysed in RIPA buffer (Cell Signaling Technology) supplemented with protease/phosphatase inhibitor cocktail (Cell Signaling Technology). Proteins from the samples were separated by SDS-PAGE and then transferred onto the polyvinylidene difluoride (PVDF) membrane (Millipore). After blocking the membrane with BSA, immunoblotting was performed using the specified primary antibodies against NLRP3 (Proteintech, 68102-1-Ig), cleaved caspase-1 (Affinity, AF4005), GSDMD (Affinity, AF4012), IL-1β (Abcam, ab283818), IL-18 (Proteintech, 10663-1-AP), eNOS (Cell Signaling Technology, 32027 S), and VE-cadherin (Abcam, ab33168). HRP-conjugated secondary antibodies (Abcam) were used to detect primary antibodies. The immunoreactive bands were visualized using enhanced chemiluminescence kit (Millipore).

### Wound healing assay

Endothelial cells were seeded in 6-well plates and cultured until a confluent monolayer formed. The cells were then maintained in serum-free medium for 24 h of starvation. A sterile 200 µL pipette tip was used to create a scratch wound. The cells were then co-cultured with conditioned medium from macrophages subjected to various treatments for 24 h. Specifically, macrophages were treated with LPS and ATP in the stimulation group, pre-treated with PTX before ATP in the PTX group, and pre-treated with DSF before ATP in the DSF group. Conditioned medium was defined as the supernatants collected from treated macrophages after removing the original medium and adding fresh medium. Images were acquired at 0 h and 24 h post-scratch. The percentage of cell migration distance was quantified using ImageJ software (NIH). The experiments were performed independently in triplicate.

### Transwell assay

Vascular smooth muscle cells were resuspended into the single-cell suspension using serum-free medium. Cells were seeded at a density of 1 × 10^4^ cells per well into the upper chambers of transwell plates (aperture 8.0 μm). Conditioned medium collected from differently treated macrophages were added to the lower chambers. After 24 h of incubation with the smooth muscle cells, the cells on the upper surface were wiped off, and the migrated cells on the lower surface were fixed with 4% paraformaldehyde and stained with 0.5% crystal violet. Images were captured using an optical microscope (Leica Microsystems) and the number of migrated cells was quantified using Image J software. Independent experiments were conducted three times.

### Co-culture of CM, ECs, and SMCs

To investigate the growth behavior of endothelial cells and smooth muscle cells, both cell types were co-cultured with conditioned medium derived from treated macrophages. The treatment of macrophages was performed as described in the wound healing assay. ECs and SMCs were labeled with the live-cell tracers green CMFDA and orange CMTMR, respectively. The labeled ECs and SMCs were suspended in the conditioned medium at a density of 1 × 10^5^ cells/mL and added to the 6 well plate in equal volumes. After 24 h of incubation, the growth of the cells was observed using the inverted fluorescence microscope. The number of adherent cells was quantitatively analyzed with ImageJ software to calculate the adhesion ratio of ECs to SMCs.

### Statistical analysis

Data are presented as mean ± standard deviation (SD). Statistical analysis was performed using GraphPad Prism software (version 10.0). Differences between two groups were assessed using the Student’s t-test. Significant differences among multiple groups were determined using one-way analysis of variance (ANOVA), followed by Tukey’s post hoc test. *P* < 0.05 was considered statistically significant.

## Discussion

Endovascular intervention, while restoring patency to occluded vessels, exert mechanical injury on the vascular wall. This injury instigates a localized inflammatory response in the affected vessels, leading to endothelial dysfunction and subsequent excessive proliferation of SMCs [6, 12, 83, 84]. Clinically, as improvements over bare-metal stents or balloons, vascular devices coated with antiproliferative agents have demonstrated poor long-term (36 months, limited elution period) patency rates and high thrombosis rates, accompanied by increased frequencies of reintervention and amputation [9–11, 13, 85]. The antiproliferative agents within the coatings inhibit cellular proliferation, and their cytotoxicity not only fails to alleviate the pre-existing inflammation but instead exacerbates it [6, 12]. In preclinical studies, anti-inflammatory strategies have been explored to inhibit restenosis [86–88]. However, challenges such as limited efficacy, non-negligible side effects, and the complexity of preparation have hindered their clinical translation [86, 87, 89].

Although inflammation is closely associated with the development and progression of restenosis, previous studies mainly focused on anti-inflammatory strategies targeting ECs and SMCs, demonstrating limited treatment effectiveness [88, 90–92]. Here, we describe the single-cell transcriptomic landscape of VBI rat arteries and identify monocytes/macrophages as the primary cell type involved in the inflammatory response. Furthermore, in contrast to the minimal presence of macrophages in the control group, a substantial population of newly emerged monocytes/macrophages in restenotic vessels undergoes pyroptosis. These findings highlight the dominant role of macrophages in post-vascular intervention inflammation and may explain the limited efficacy of strategies targeting ECs and SMCs.

Previous research indicates that sex may influence the occurrence and progression of restenosis [93, 94]. As investigating sex-related disparities in restenosis was not the primary focus of the current study, only male rats were utilized. The potential impact of sex on macrophage pyroptosis-driven restenosis will be further investigated in future studies. We demonstrate that GSDMD-mediated macrophage pyroptosis promotes vascular inflammation and restenosis following endovascular intervention. Previous studies have shown that levels of IL-1β and IL-18 are significantly elevated in patients with vascular restenosis [25–27]. Although these studies indicate an association between macrophage pyroptosis and restenosis after endovascular intervention, much of the work was associative. Injured arteries with specific knockdown of macrophage GSDMD show inhibition of pyroptotic inflammation, promotion of re-endothelialization, and alleviation of neointimal hyperplasia. Therefore, this study provides a theoretical basis for anti-restenosis therapeutic strategies targeting GSDMD-mediated pyroptosis.

Based on the discovered mechanism, the pyroptosis inhibitor DSF, which directly targets GSDMD pore formation [32], logically emerges as a candidate therapeutic agent against restenosis. Although not previously applied in restenosis research, DSF has been used in long-term clinical practice as an anti-alcohol addiction agent with established safety, suggesting that its application in restenosis may not result in significant clinical toxicity [45, 95]. Furthermore, our study found that key components of multiple inflammasomes are primarily localized in monocytes/macrophages, implying that inhibitors targeting only a single inflammasome have limited efficacy due to compensatory activation of other inflammasomes. DSF targets the common downstream effector GSDMD of inflammasome activation, potentially suppressing inflammation resulting from all inflammasome activations [32, 96], which is the key reason we chose it.

DSF is rapidly metabolized and possesses high hydrophobicity and lipophilicity, factors that constrain its systemic application [56, 97, 98]. Therefore, we devised DSF-coated balloons (DCB) to directly deliver DSF into the injured vessel wall and enhance its local accumulation, inhibiting pyroptosis and blocking the release of pro-inflammatory cytokines. Although polymers can enhance the loading of therapeutic agents, they may induce inflammation and compromise delivery efficiency [54, 55, 99–101]. Hence, we tested the loading of DSF on balloon surfaces without polymer use. The dip coating procedure, a widely adopted technique in the industry for balloon coating, was employed to achieve surface loading on the balloons [57]. Based on previously reported functionally effective doses of DSF for inhibiting pyroptosis and widely validated procedures for paclitaxel-coated balloons, we prepared the coating using a 5 mg/mL DSF solution [69]. In fact, taking advantage of the lipophilicity of disulfiram, we successfully developed a polymer-free, crystalline DSF-coated balloon. The DCB facilitates precise local drug delivery. Analogous to PTX-coated balloons, where the drug load is substantially lower than systemic chemotherapy doses [102–104], the amount of DSF loaded onto the DCB in this study is several orders of magnitude lower than the FDA-approved oral doses used for routine clinical therapy (125–500 mg/day). Even in the event of minimal drug wash-off into the systemic circulation, prior studies of PTX-coated balloons have shown that post-inflation plasma drug concentrations are typically extremely low or even undetectable [105, 106]. Consequently, the risk of systemic exposure is considered negligible [106–108]. Furthermore, multiple previous studies have evaluated DSF using systemic administration at doses substantially higher than those employed in the present study in cardiovascular and other disease models, without evident safety concerns [32, 42, 44, 109].

Compared to the amorphous, a crystalline coating more effectively transfers to the vessel wall via balloon dilation and establishes drug depots, enabling sustained local concentrations through gradual dissolution [58–60]. Endovascular intervention not only transfers microparticles into the vessel wall through compression and friction but also disrupts the endothelial barrier, enhancing the deep vascular penetration of DSF microparticles [59, 110]. From this perspective, the drawback of endothelial barrier disruption is transformed into an advantage for DSF delivery. Characterization of both the coated balloon and the vessel confirm substantial accumulation of DSF crystalline particles in the injured vascular wall. Finally, the strong lipophilicity of DSF facilitates its dissolution and absorption upon contact with vascular cells [56, 111, 112]. Cells can uptake DSF through nonspecific lipid exchange or endocytic mechanisms [111]. Although the locally delivered dose used in this study is extremely low, making systemic accumulation of DSF or its metabolites unlikely, future studies are still warranted to perform comprehensive pharmacokinetic and toxicokinetic evaluations of DCB, including measurements of plasma drug/metabolite levels and tissue distribution, to more fully characterize its systemic exposure profile, which remains a limitation of the current study. Furthermore, while the primary objective of this study was to elucidate the underlying biological mechanisms and validate the feasibility of the mechanism-based therapeutic strategy, rather than to complete a comprehensive device-engineering evaluation, future studies will incorporate the ultrasonic atomization spray-coating technique to further optimize the balloon coating process. This will enable more systematic assessment of coating uniformity, accelerated stability, and DSF release post-deployment [113].

We investigated the regulation of macrophage pyroptosis by the DCB in injured arteries following vascular intervention. Although PCB can transiently suppress neointimal hyperplasia, patency rates decline sharply once late-stage PTX release is exhausted, as the cytotoxicity of PTX exacerbates vascular inflammation [9, 114]. However, DCB reduced macrophage infiltration, inhibited macrophage pyroptosis, and alleviated inflammation in injured arteries, representing a fundamental departure from the mechanism of PCB. We further validated the effects of DCB on restenosis, revealing functional advantages including promoting functional endothelial repair and inhibiting neointimal hyperplasia. The risk of late-stage thrombosis remains a primary concern for all angioplasty devices and is one of the main limitations in the clinical use of drug-coated balloons [84, 115]. Here, treatment with PCB led to excessive fibrin accumulation and thrombosis in the vessel wall of a rat model. In contrast, DCB treatment reduced fibrin levels in the vessel wall, indicating a lower risk of thrombosis. Moreover, histological examination of major organs confirmed that DSF, at the therapeutic dose used on coated balloons, exhibited no cytotoxicity.

The vascular environment in vivo is inherently complex [16, 116]. While DSF was selected to target GSDMD-mediated macrophage pyroptosis, the possibility of DSF directly affecting ECs and VSMCs cannot be excluded. We further demonstrate that DSF promotes functional endothelial repair and inhibits excessive migration and proliferation of SMCs by suppressing macrophage pyroptosis. This provides new evidence for the differential interactions between macrophages and ECs/SMCs within the vascular injury microenvironment. In addition, although our data support a critical role for GSDMD-mediated macrophage pyroptosis in vascular restenosis, other regulated inflammatory cell death pathways, such as RIPK1-dependent necroptosis or GPX4-regulated ferroptosis, may also contribute to vascular injury responses and warrant further investigation in future studies.

## Conclusions

The current study reports that GSDMD-mediated macrophage pyroptosis after endovascular intervention promotes inflammation and restenosis. DSF targeting GSDMD pore formation has been designed into balloon coating, achieving precise delivery to the injured vessel wall via balloon dilation. DCB inhibits macrophage pyroptosis and associated inflammation by blocking GSDMD pore formation, promoting functional re-endothelialization and suppressing neointimal hyperplasia, thereby effectively preventing long-term vascular restenosis. Future studies are warranted to further evaluate the systemic safety profile and perform comprehensive pharmacokinetic validation of DCB in large-animal models prior to clinical translation. Due to limitations at active sites, it is not feasible to directly conjugate fluorescent probes to DSF to observe its intracellular uptake. Therefore, we consider utilizing nanocarriers to actively target macrophages and achieve visualization of DSF within cells in future. Taken together, our study highlights GSDMD-mediated macrophage pyroptosis as a novel therapeutic target against vascular restenosis. The customed DCB based on this mechanism, as a drug-coated device that inhibits pyroptosis with a favorable biosafety profile, provides a promising strategy for vascular restenosis prevention.

## Supplementary Information

Below is the link to the electronic supplementary material.

Supplementary Material 1

## Data Availability

The data that support the findings of this study are available from the corresponding author upon reasonable request.

## Abbreviations

CM: Conditioned medium
CVD: Cardiovascular disease
DCB: Disulfiram-coated balloon
DEG: Differentially expressed gene(s)
DSF: Disulfiram
EC/ECs: Endothelial cell(s)
GSDMD: Gasdermin D
GSDMD-NT: N-terminal fragment of gasdermin D
I/M: Intima-to-media ratio
OSP: Optical surface profilometry
PCB: Paclitaxel-coated balloon
PTX: Paclitaxel
VBI: Vascular balloon injury
VSMC/VSMCs: Vascular smooth muscle cell(s)
