# Supplementary material for: Macrophage pyroptosis inhibition alleviates postinjury neointimal formation and vascular restenosis
Source: J Transl Med. 2026 Feb 3;24:315. doi: 10.1186/s12967-026-07777-z (PMC12958643; doi:10.1186/s12967-026-07777-z)
Supplement: Supplementary file 1 — Supplementary Material 1 [file 12967_2026_7777_MOESM1_ESM.docx]

**Supporting Information**

**Macrophage pyroptosis inhibition alleviates postinjury neointimal formation and vascular restenosis**

Zaixiong Ji, Meijuan He, Hong Wu, Shixiong Chen, Suhe Wang, Xiaorui Yin, Han Wang^*^


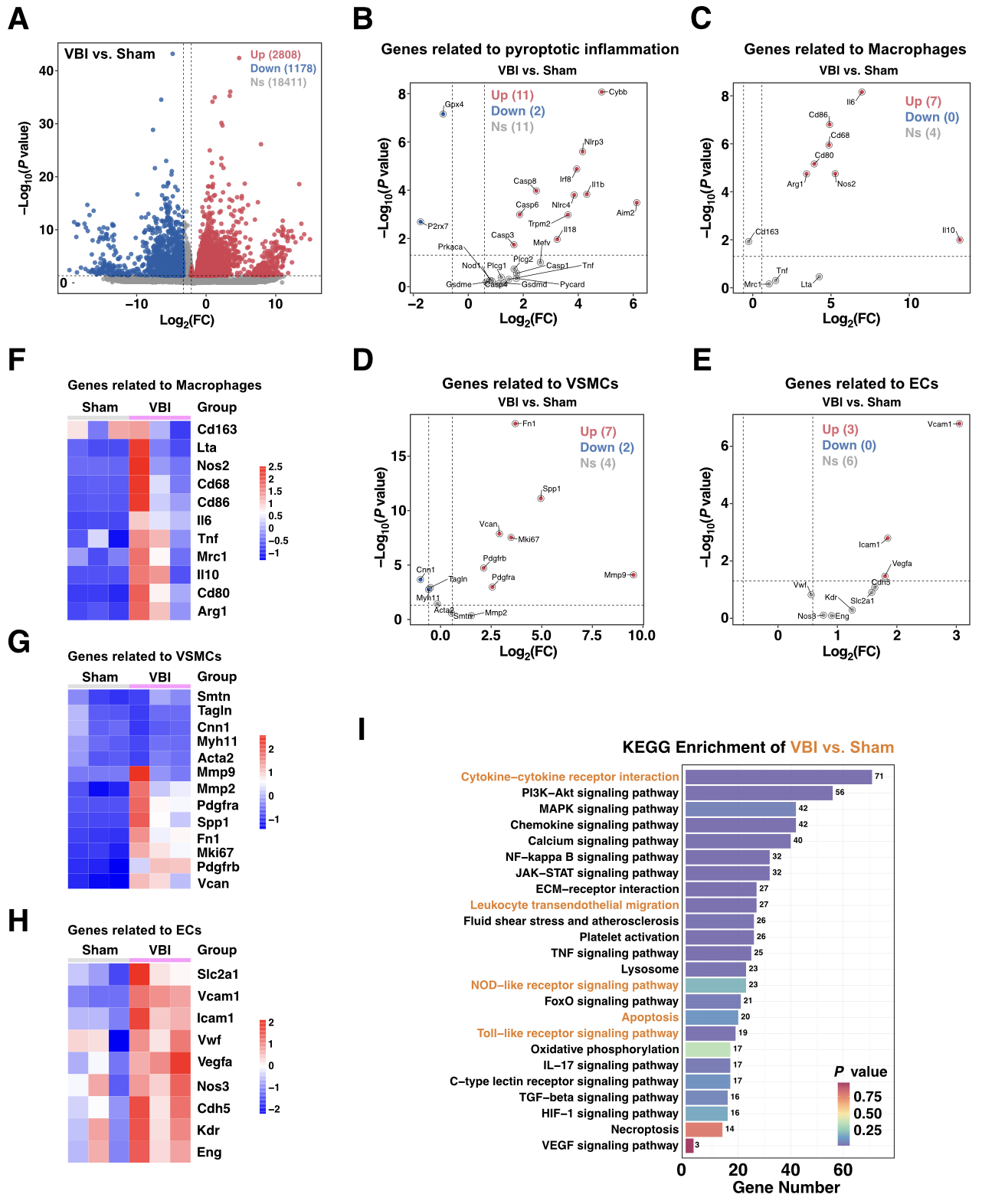


**Figure S1.** (A) Volcano plot showing differentially expressed genes in the VBI group compared to the Ctrl (Sham) group. (B) Volcano plot showing differentially expressed genes related to pyroptotic inflammation between the VBI group and Ctrl group. (C) Volcano plot showing differentially expressed genes related to macrophage between the VBI group and Ctrl group. (D) Volcano plot showing differentially expressed genes related to VSMC between the VBI group and Ctrl group. (E) Volcano plot showing differentially expressed genes related to EC between the VBI group and Ctrl group. (F) Heatmap showing genes related to macrophage in the Sham group and VBI group. (G) Heatmap showing genes related to VSMC in the Sham group and VBI group. (H) Heatmap showing genes related to EC in the Sham group and VBI group. (I) Kyoto Encyclopedia of Genes and Genomes (KEGG) pathway enrichment analysis of differentially expressed genes between the VBI group and the Sham group. (Significantly differentially expressed genes were defined with |fold-change| > 1.5 and P < 0.05).


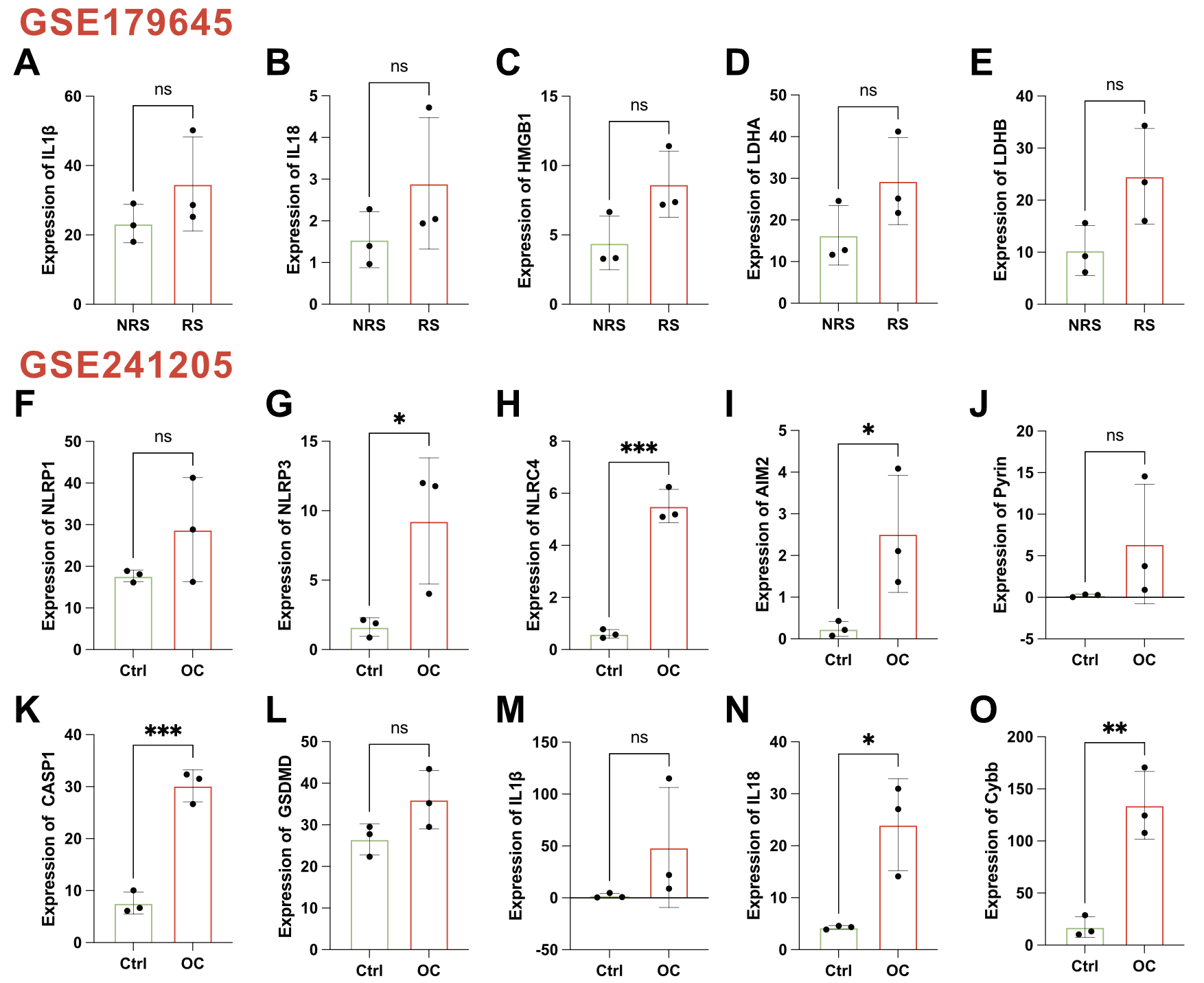


**Figure S2.** (A-E) Re-analysis of the microarray dataset (GSE179645) showing the increased expression levels of IL1b, IL18, HMGB1, LDHA, LDHB in the blood of patients with restenosis after vascular interventions. (F-O) RNA-seq analysis of normal veins (Ctrl) and occluded vein grafts (OC) (GSE241205) showing a trend of increased expression levels of key pyroptosis-related genes in occluded vein grafts.

Data are presented as mean ± standard deviation (SD). Statistical signiﬁcance was determined by one-way ANOVA with Tukey’s test. *P < 0.05, **P < 0.01, ***P < 0.001. ns, no signiﬁcant difference.


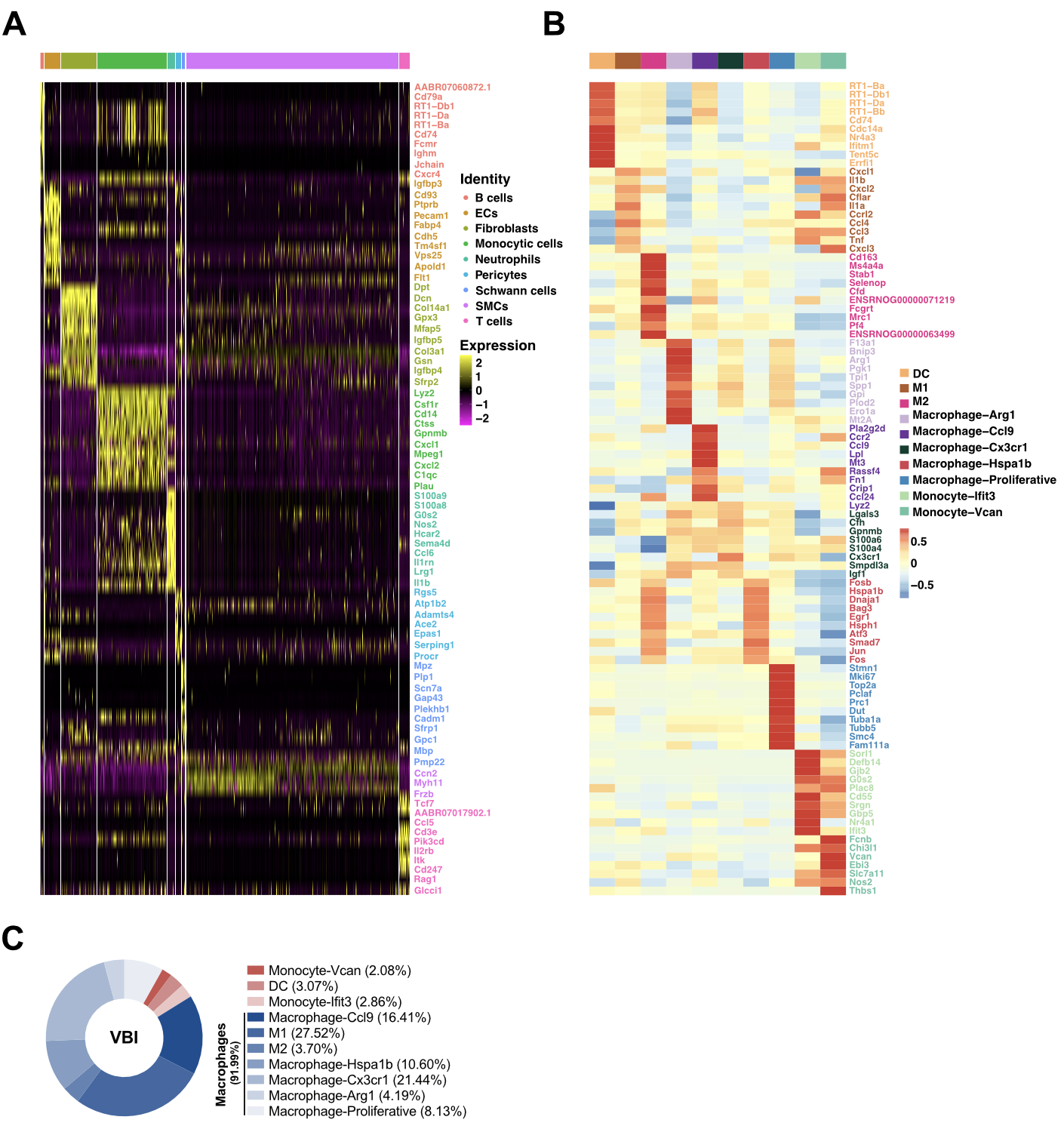


**Figure S3.** (A) Heatmap shows the expression of marker genes identifying cell populations. (B) Heatmap identifying markers of monocytic subpopulations. (C) The proportion of each monocytic subpopulation in the VBI group.


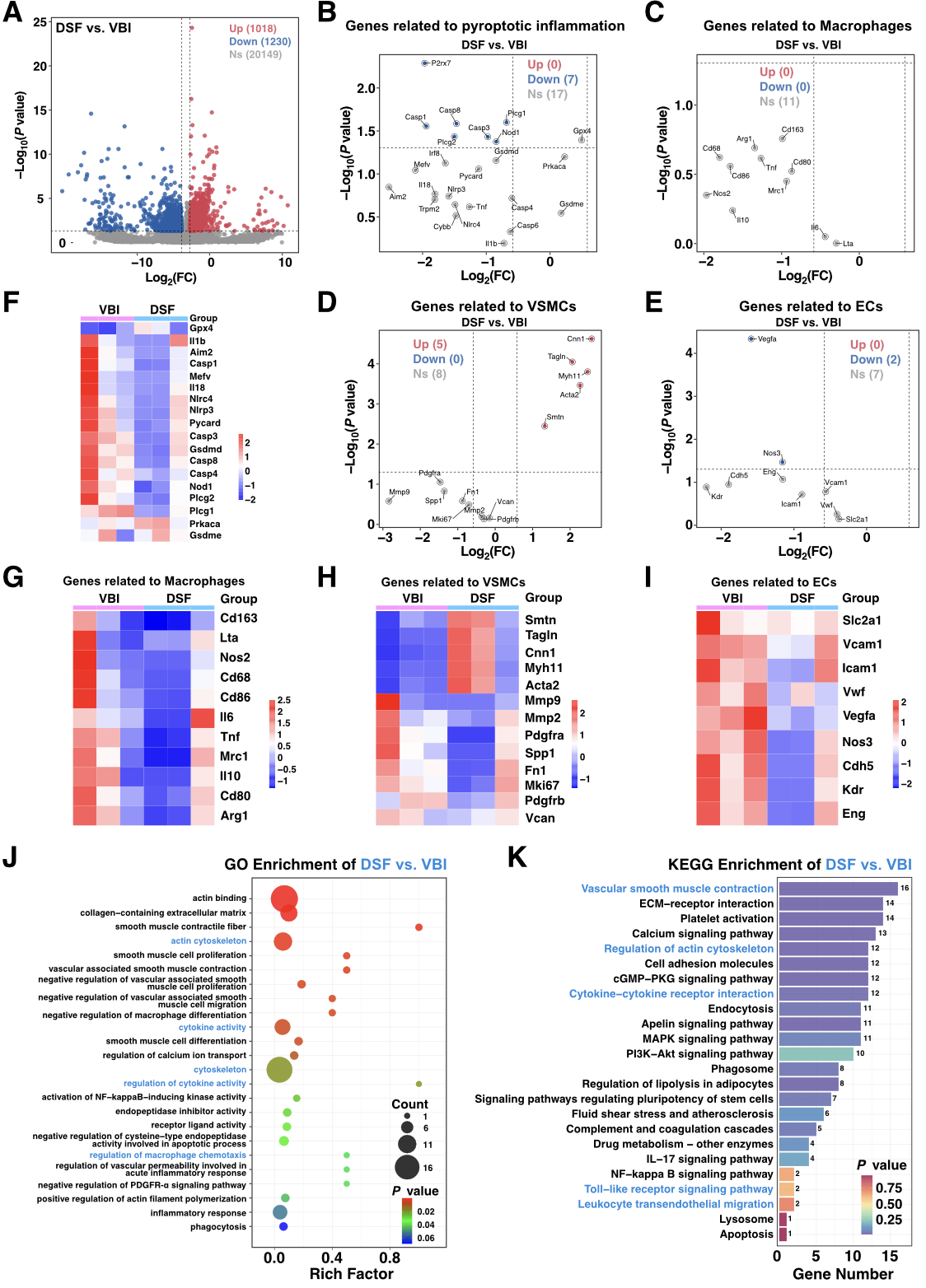


**Figure S4.** (A) Volcano plot showing differentially expressed genes in the VBI group compared to the DSF group. (B) Volcano plot showing differentially expressed genes related to pyroptotic inflammation between the DSF group and VBI group. (C) Volcano plot showing differentially expressed genes related to macrophage between the DSF group and VBI group. (D) Volcano plot showing differentially expressed genes related to VSMC between the DSF group and VBI group. (E) Volcano plot showing differentially expressed genes related to EC between the DSF group and VBI group. (F) Heatmap showing genes related to pyroptotic inflammation in the VBI group and DSF group. (G) Heatmap showing genes related to macrophage in the VBI group and DSF group. (H) Heatmap showing genes related to VSMC in the VBI group and DSF group. (I) Heatmap showing genes related to EC in the VBI group and DSF group. (J) Gene Ontology (GO) analysis of differentially expressed genes between the DSF group and the VBI group. (K) KEGG pathway enrichment analysis of differentially expressed genes between the DSF group and the VBI group.


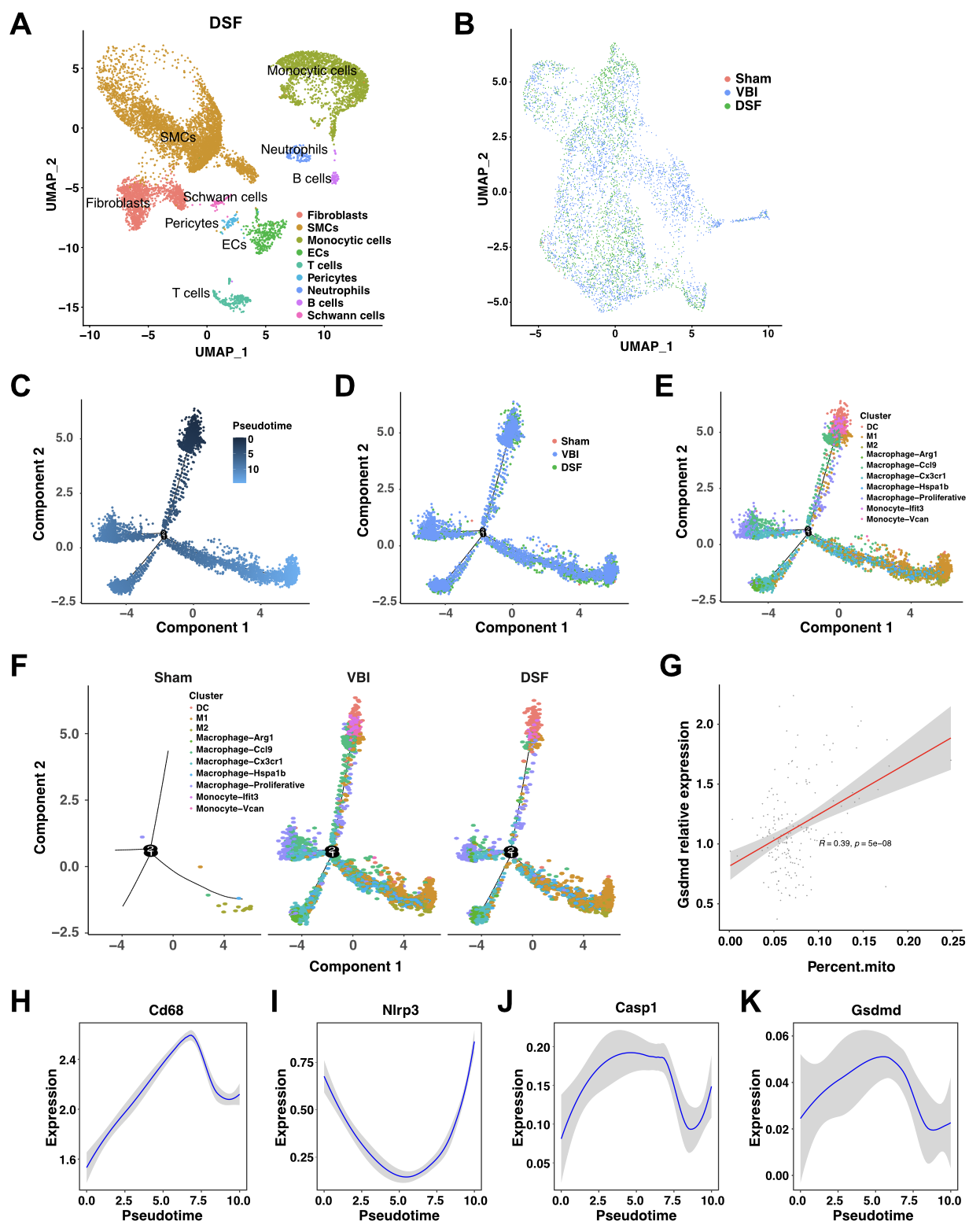


**Figure S5.** (A) UMAP plot of cell populations from the DSF group in rat common carotid arteries. (B) UMAP plot showing monocytic populations in the Sham group, VBI group, and DSF group. (C-E) Biaxial scatter plots visualizing the developmental trajectory of monocytic populations. The color gradient represents developmental stages, with dark colors indicating early development. (F) Pseudotime developmental analysis showing the single-cell trajectory branching from dendritic cells and monocytes in the Sham group, VBI group, and DSF group. (G) Correlation analysis between GSDMD expression and mitochondrial gene content. The red line represents the linear regression mean, and the shaded area represents the 95% confidence interval. (H-J) Pseudotime developmental analysis of Cd68, Nlrp3, and Casp1 showing their changes throughout the process. (K) Pseudotime developmental analysis of GSDMD showing changes throughout the process.


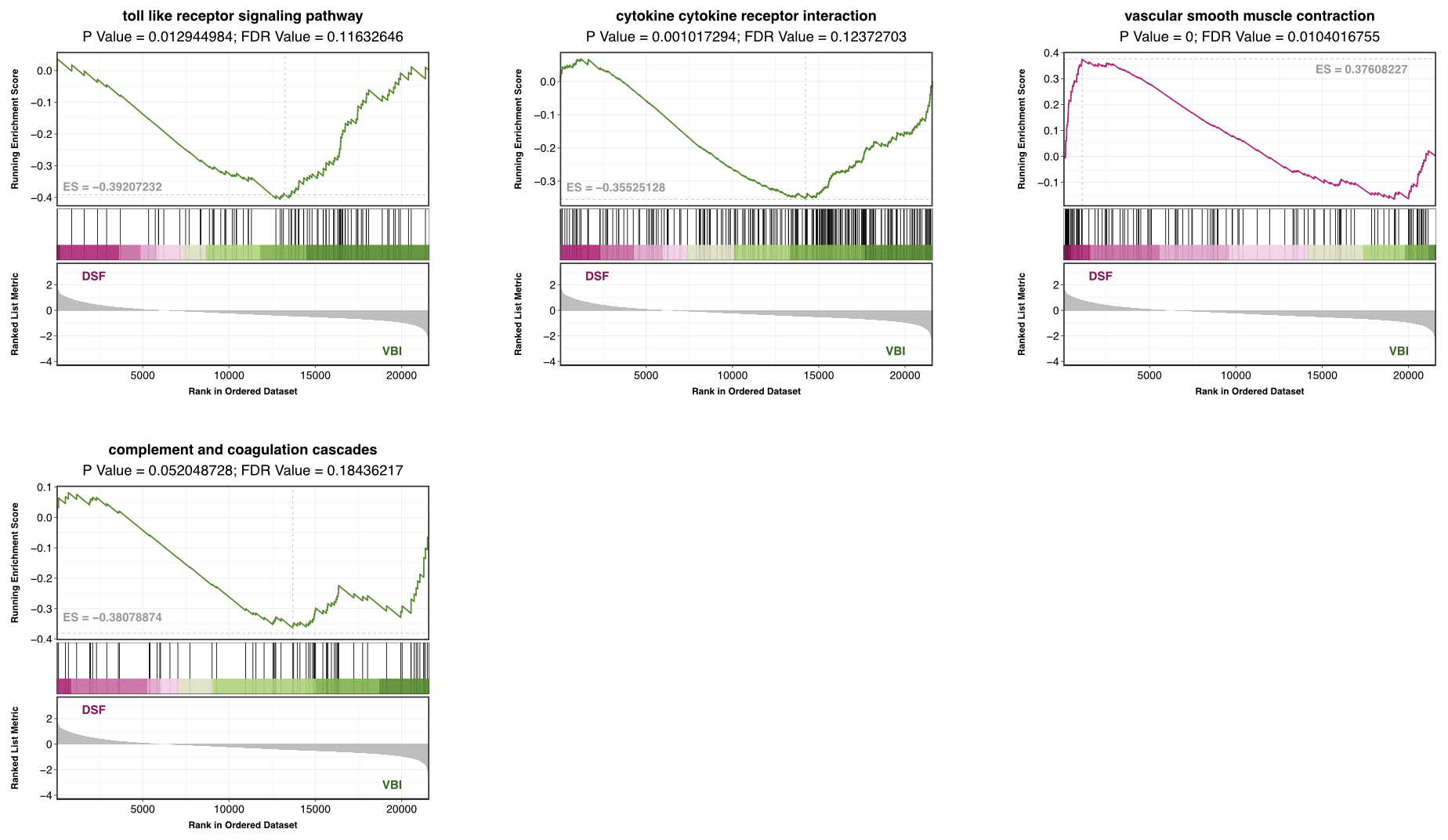


**Figure S6.** The Gene Set Enrichment Analysis (GSEA) of differentially expressed genes between the DSF group and the VBI group based on KEGG pathways.


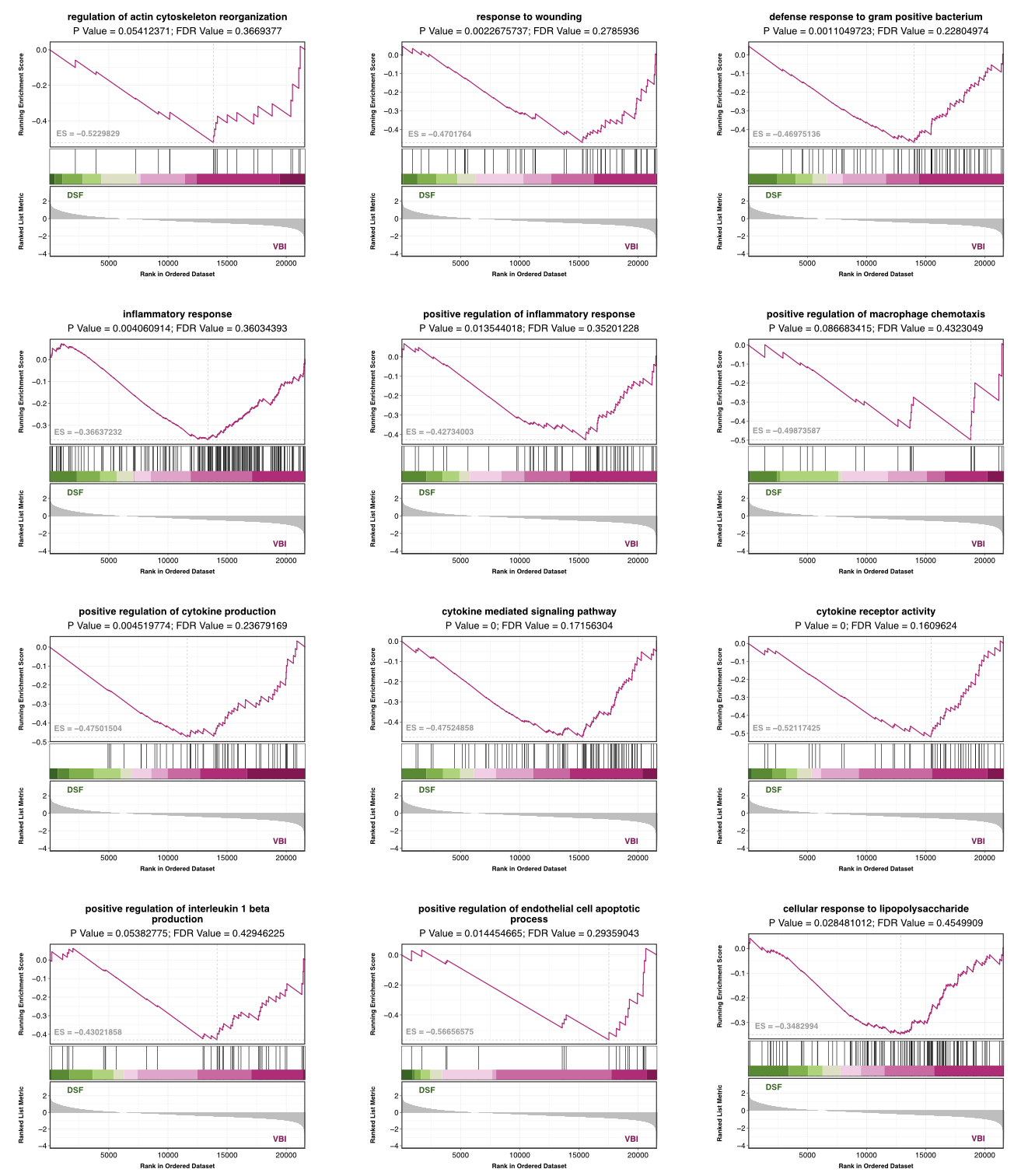


**Figure S7.** The Gene Set Enrichment Analysis (GSEA) of differentially expressed genes between the DSF group and the VBI group based on Gene Ontology (GO).


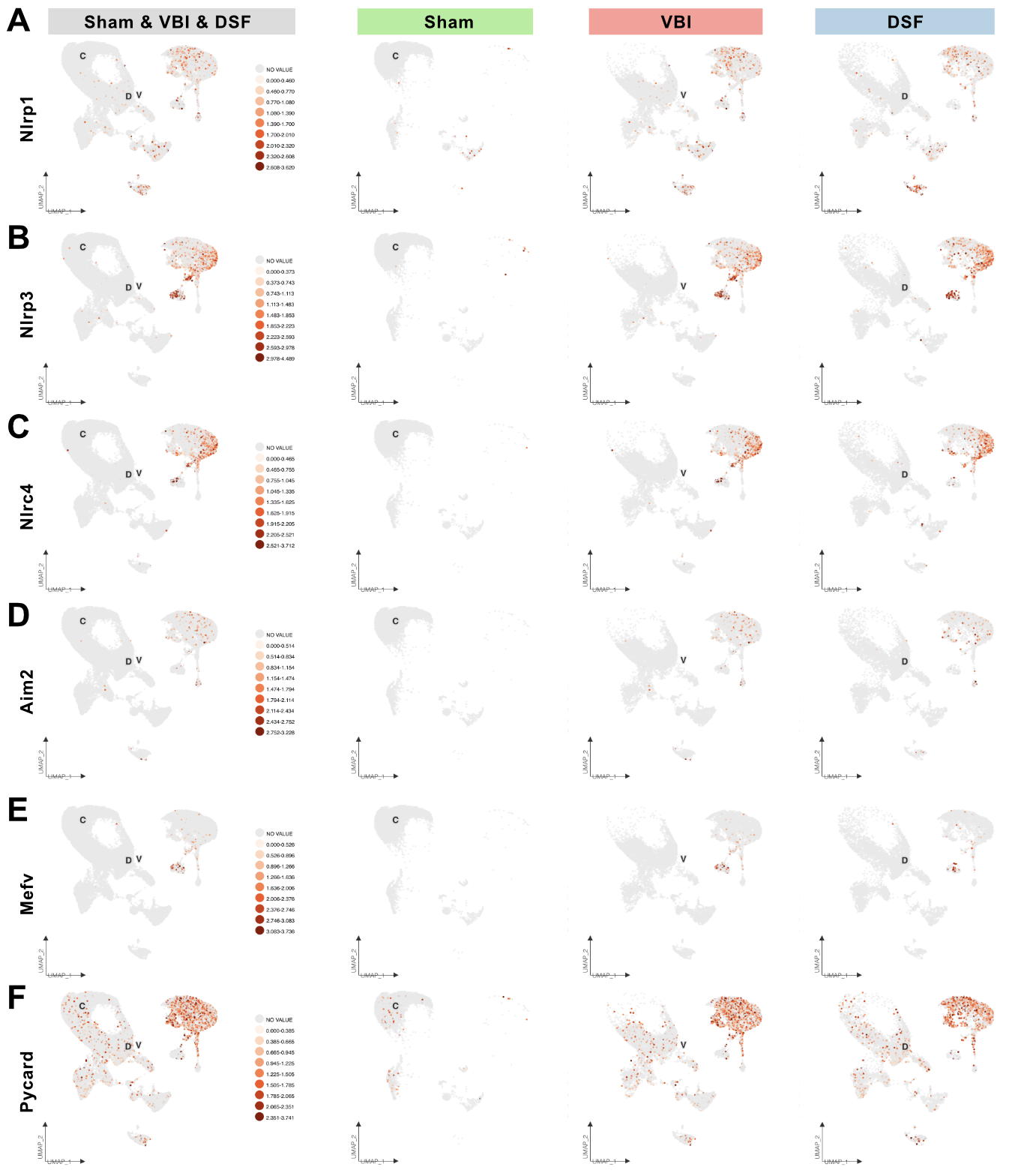


**Figure S8.** Biaxial scatter plots showing the distribution and expression of Nlrp1, Nlrp3, Nlrc4, Aim2, Mefv, and Pycard across all cell populations in the Sham group, VBI group, and DSF group.


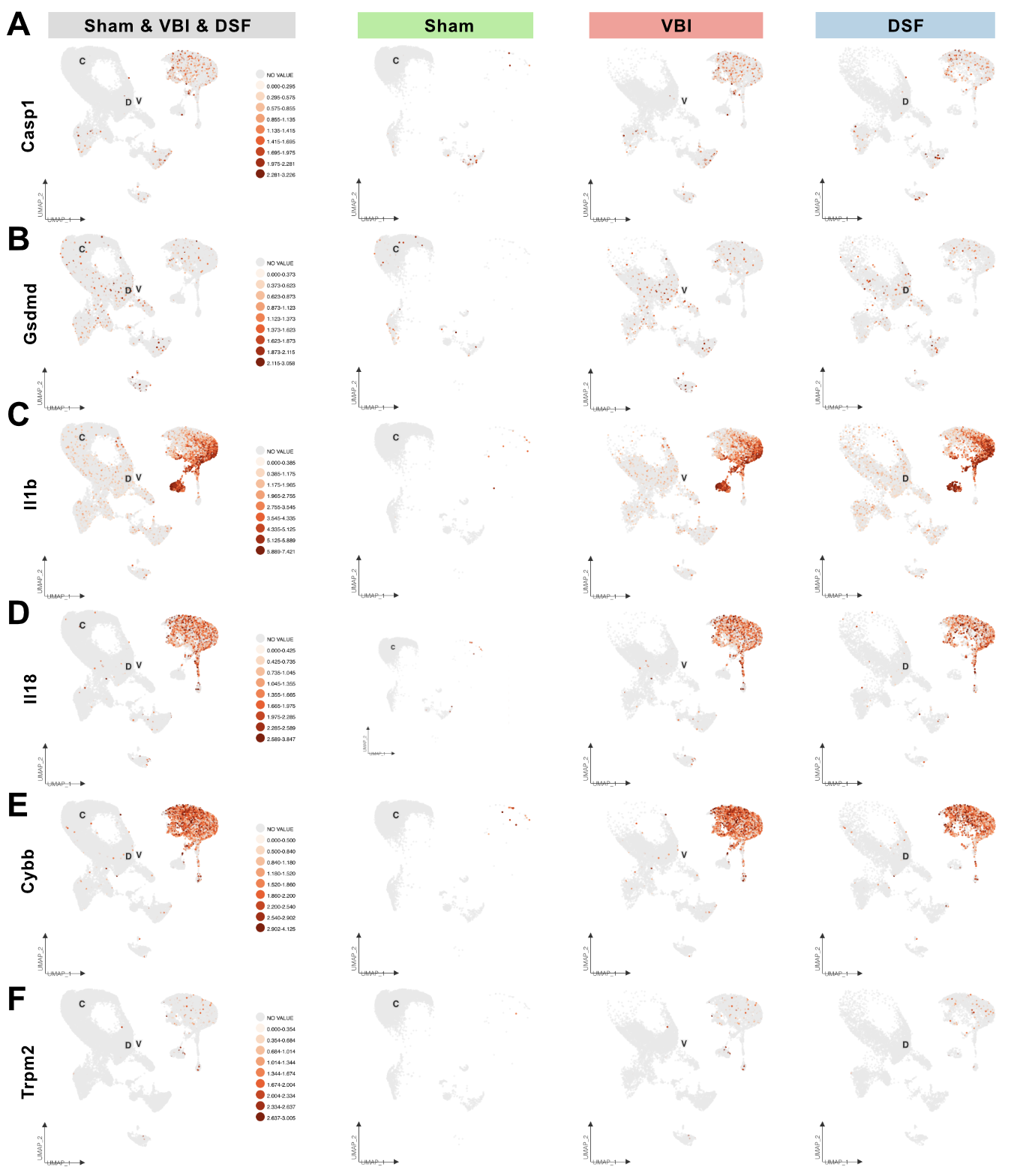


**Figure S9.** Biaxial scatter plots showing the distribution and expression of Casp1, Gsdmd, Il1b, Il18, Cybb, and Trpm2 across all cell populations in the Sham group, VBI group, and DSF group.


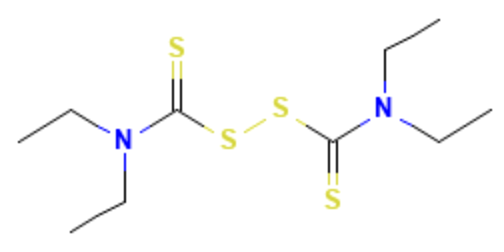


**Figure S10.** Chemical structure depiction of disulfiram was obtained from PubChem compound (PubChem CID: 3117).


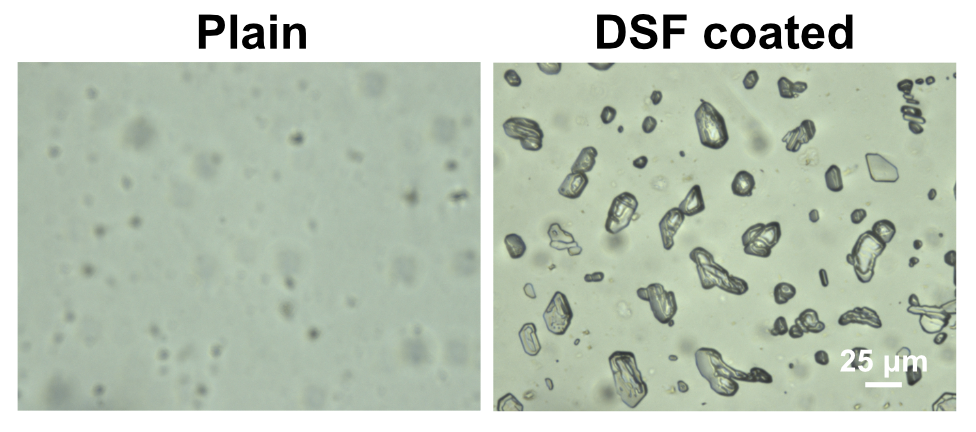


**Figure S11.** Brightfield imaging of the surface morphology of unused plain balloons and DSF coated balloons.


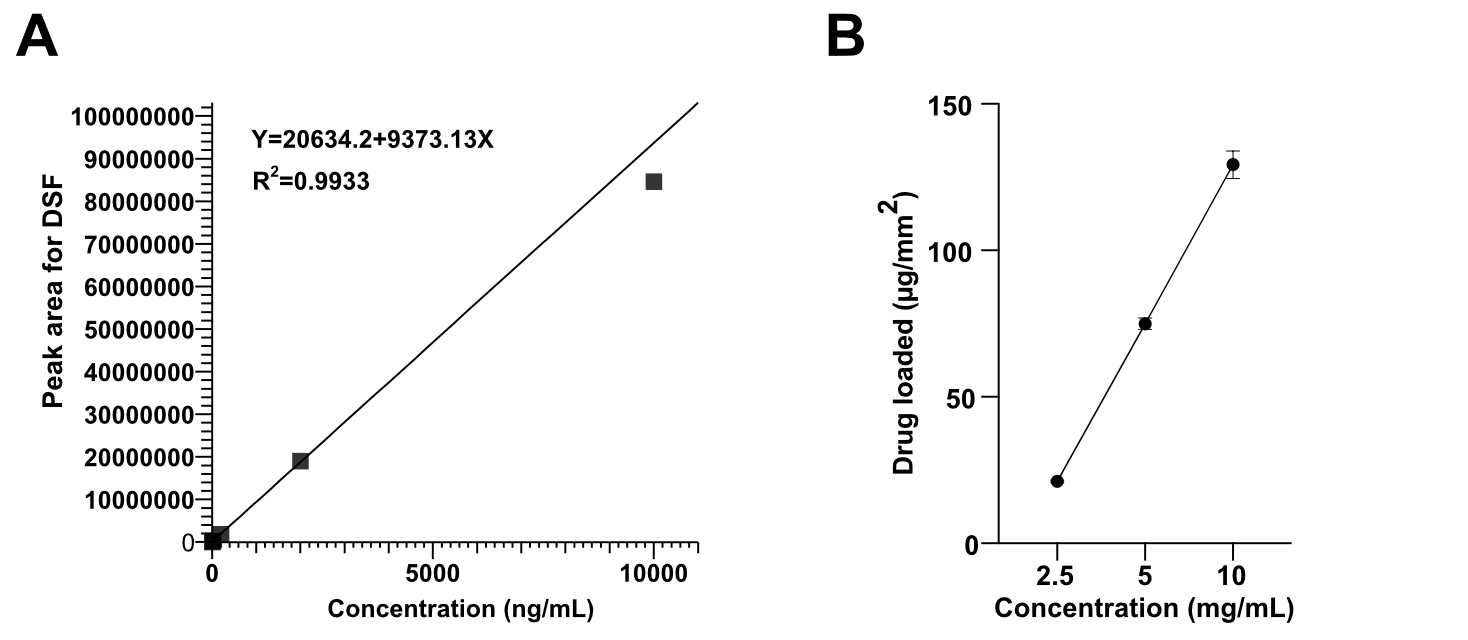


**Figure S12.** DSF standard curves by HPLC (A). Total amount of drug (μg/mm^2^) loaded in DSF coated balloon (B).


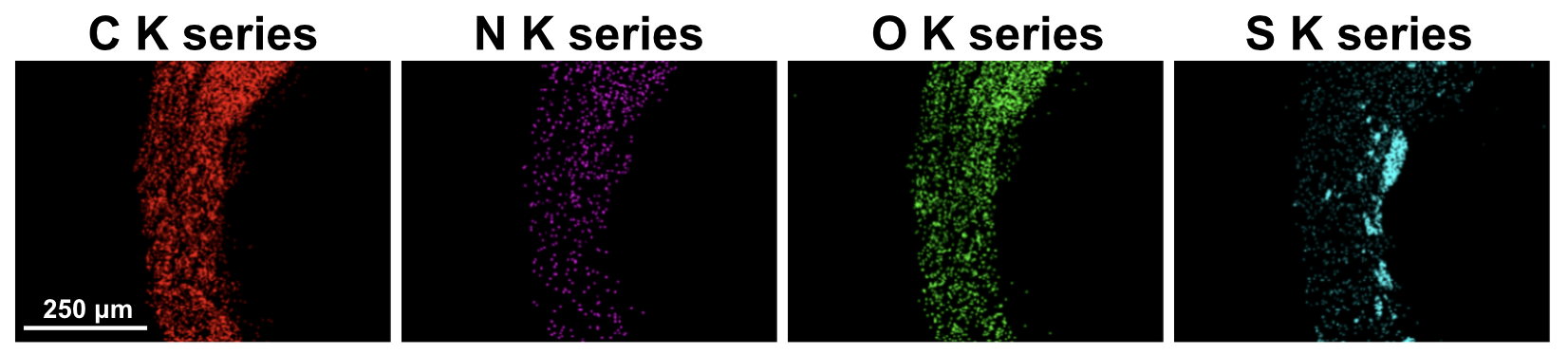


**Figure S13.** Element mapping of carotid artery treated with DSF coated balloon.


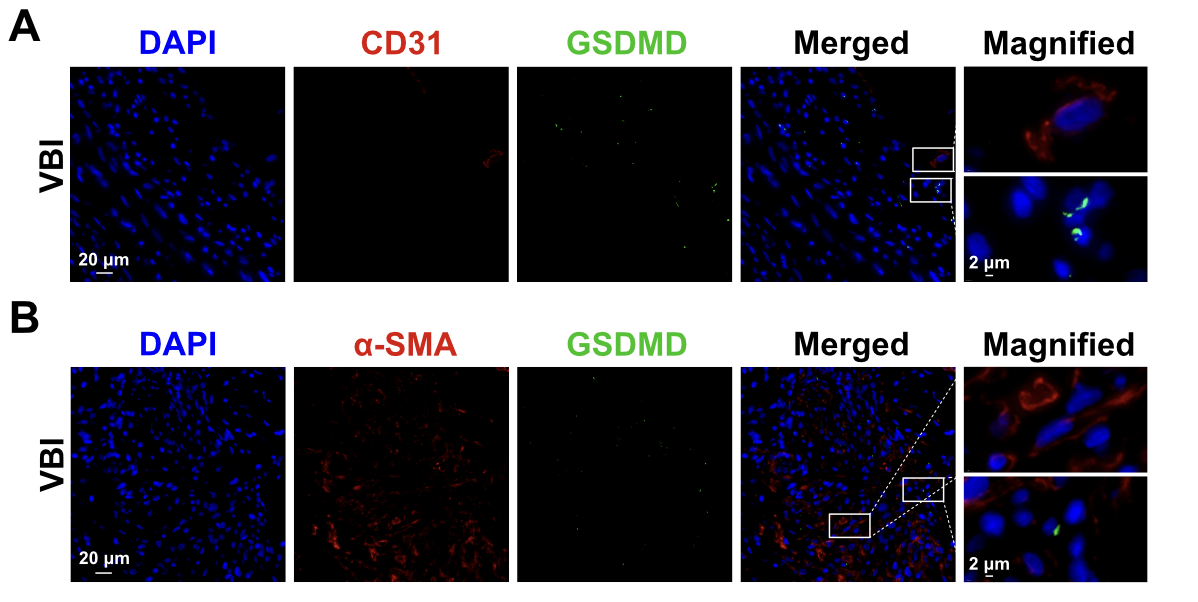


**Figure S14.** Immunoﬂuorescence imaging of CD31 and GSDMD co-staining (A) in left common carotid arteries from VBI rats (n = 6). Immunoﬂuorescence imaging of α-SMA and GSDMD co-staining (B) in left common carotid arteries from VBI rats (n = 6).


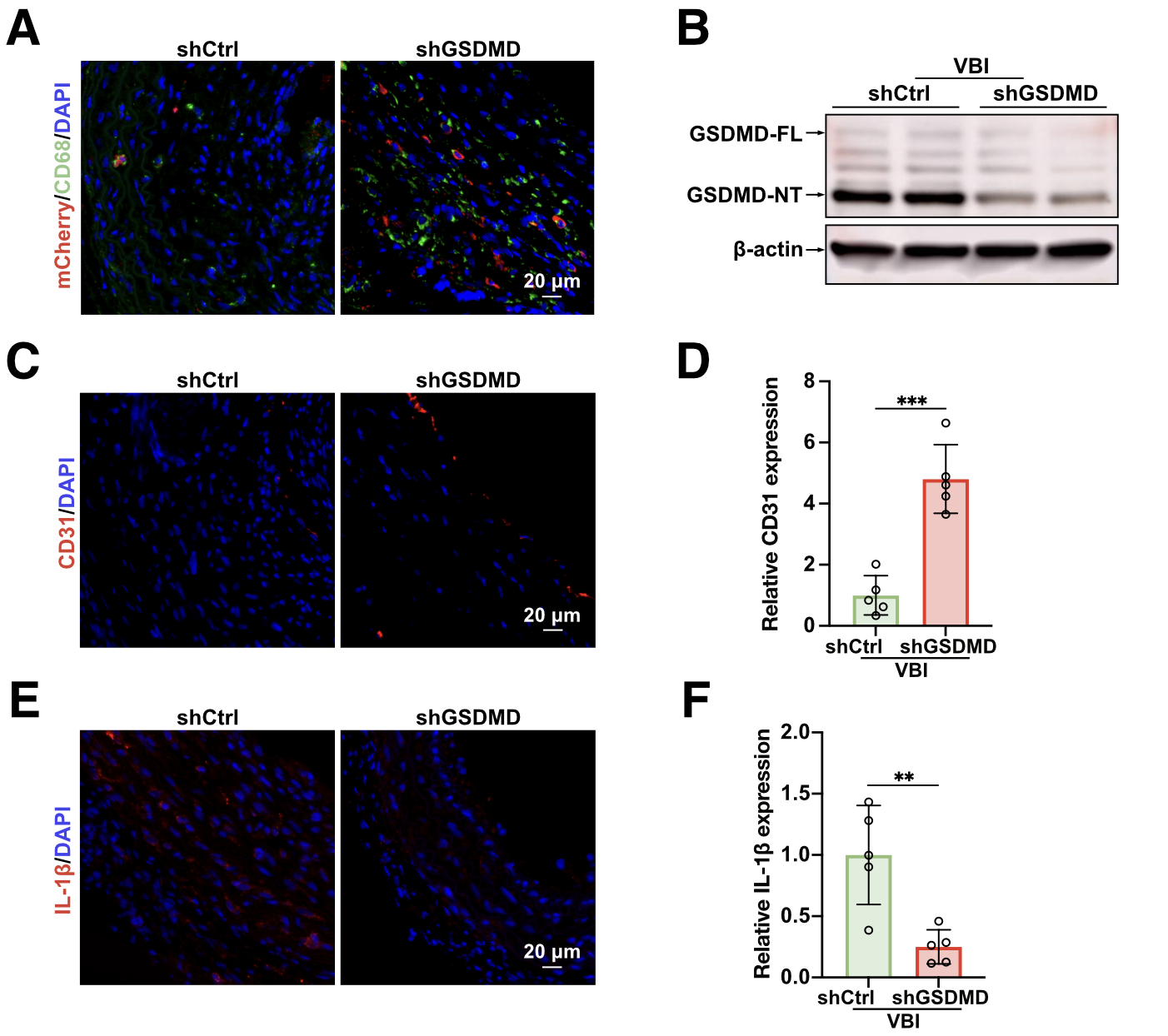


**Figure S15.** Knockdown of specific macrophage GSDMD in rats. (A) Representative images of mCherry expression (red) and CD68 (green) in the carotid arteries of VBI rats infected with shCtrl or shGSDMD. FL, full-length; NT, N-terminal. (B) Representative Western blots and quantification showing the levels of GSDMD protein in the carotid arteries of VBI rats infected with shCtrl or shGSDMD. (C) Representative images of CD31 staining showing endothelial cells in rat arteries. (D) Quantification of relative intimal CD31 expression (n = 5). (E) Representative images of IL-1β staining in rat arteries. (F) Quantification of relative intimal IL-1β expression (n = 5).

Data are presented as mean ± standard deviation (SD). Statistical signiﬁcance was determined by one-way ANOVA with Tukey’s test. **P < 0.01, ***P < 0.001. ns, no signiﬁcant difference.


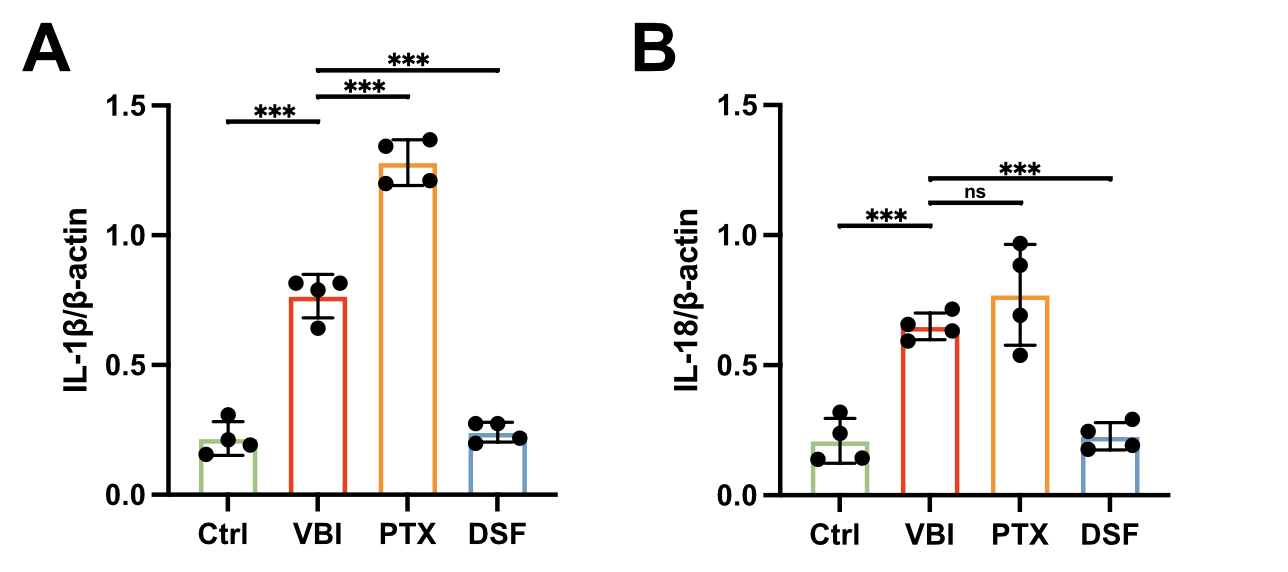


**Figure S16.** Western blotting analysis of left common carotid arteries from four indicated groups of rats at 4 weeks. Quantification of IL‐1β (A) and IL18 (B) normalized to β-actin levels (n = 4).

Data are presented as mean ± standard deviation (SD). Statistical signiﬁcance was determined by one-way ANOVA with Tukey’s test. *P < 0.05, **P < 0.01, ***P < 0.001. ns, no signiﬁcant difference.


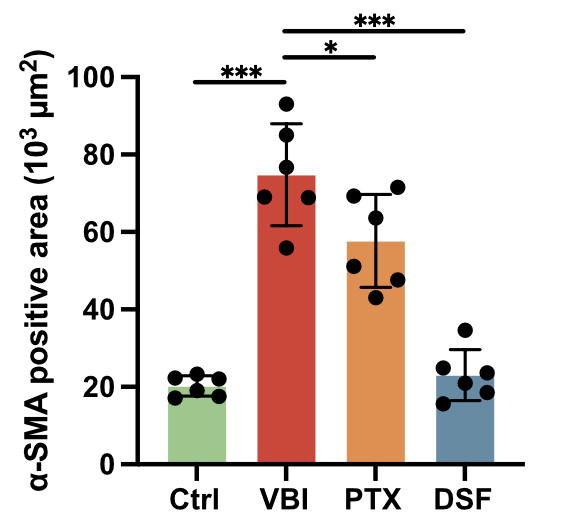


**Figure S17.** Representative immunofluorescence images of CD31 (red) and α-SMA (green) staining taken from left common carotid arteries from four indicated groups of rats at 4 weeks. Quantification of α-SMA staining (n = 6).

Data are presented as mean ± standard deviation (SD). Statistical signiﬁcance was determined by one-way ANOVA with Tukey’s test. *P < 0.05, ***P < 0.001. ns, no signiﬁcant difference.

| Group | Ctrl | VBI | PTX | DSF |
| --- | --- | --- | --- | --- |
| New Thrombus Cases (A) | 0 | 2 | 4 | 1 |
| Total Observed Population (B) | 6 | 6 | 6 | 6 |
| Thrombosis Rate (A/B * 100%) | **0.00 %** | **33.33 %** | **66.67 %** | **16.67 %** |

**Figure S18.** Thrombosis rates of the left common carotid arteries from four indicated groups of rats at 4 weeks.


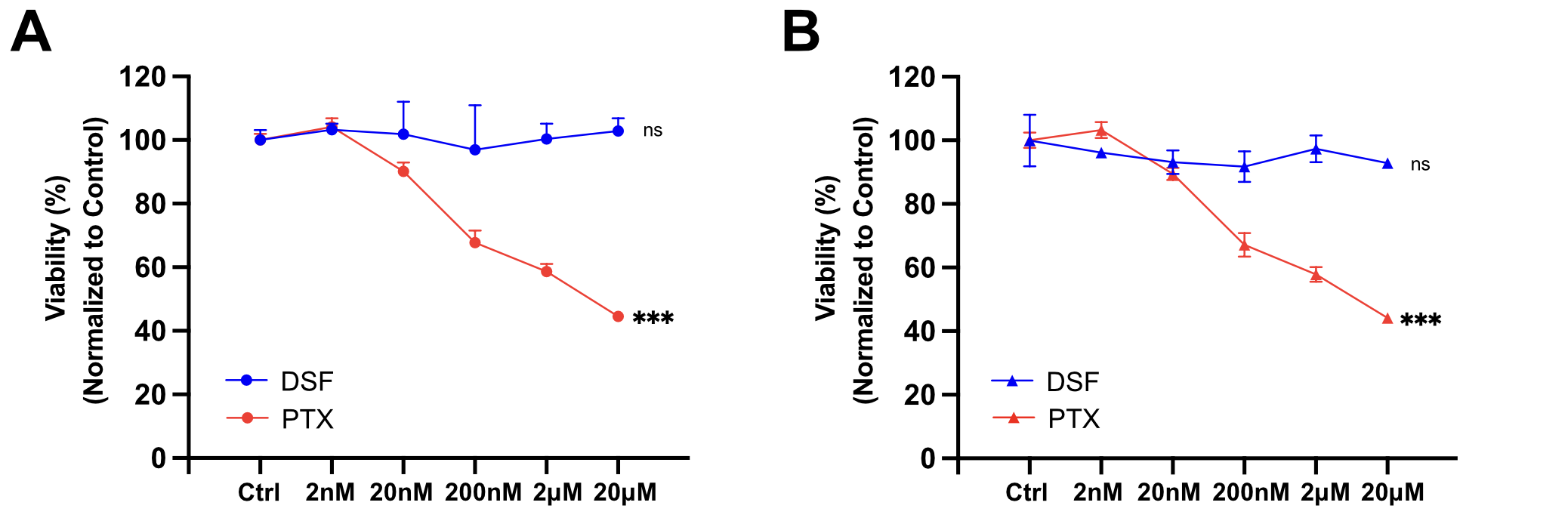


**Figure S19.** Viability of macrophages (A) and vascular smooth muscle cells (B) treated with different doses of DSF or PTX for 12 hr (n = 3).

Data are presented as mean ± standard deviation (SD). Statistical signiﬁcance was determined by one-way ANOVA with Tukey’s test. ***P < 0.001. ns, no signiﬁcant difference.


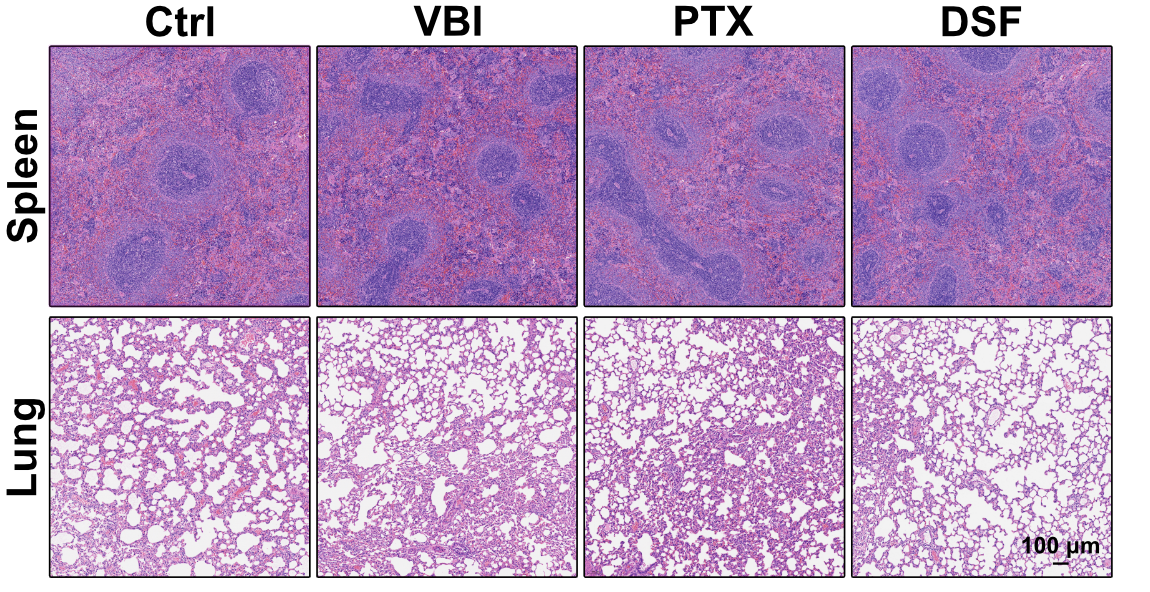


**Figure S20.** Representative H&E-stained images of major organs, including the spleen and lungs, to assess the biosafety of various treatments (n = 6).
